# Context‐Dependent Chemoselectivity of Aromatic C‐Methyltransferases

**DOI:** 10.1002/cbic.70294

**Published:** 2026-04-24

**Authors:** Juliane Breiltgens, Ziruo Zou, Sascha Ferlaino, Jennifer N. Andexer, Michael Müller

**Affiliations:** ^1^ Institute of Pharmaceutical Sciences University of Freiburg Freiburg Germany

**Keywords:** C‐methylflavonoids, Friedel–Crafts alkylation, nonproteinogenic amino acids, small molecules, tailoring reaction

## Abstract

*S*‐adenosyl‐l‐methionine (SAM)‐dependent methyltransferases (MTs) are generally classified as C‐, O‐, N‐, S‐, or halide MTs depending on their methyl acceptor. C‐MTs catalyze selective methylation reactions of carbon nucleophiles and play a crucial role in the regulation and diversification of natural products. The control of chemoselectivity by these enzymes is poorly understood, especially with respect to the resonance of a nucleophilic neighboring group that activates the carbon methylation site. We investigated two aromatic C‐MTs for the underlying mechanisms governing their chemo‐ and/or regioselectivity. The unprecedented in vitro dimethylation activity of SfmM2 and NapB5 was demonstrated using the native substrate l‐tyrosine and substrates with a 2,4‐dihydroxyacetophenone pattern, respectively. Substrate symmetry and the in situ SAM supply with removal of the competitive inhibitor *S*‐adenosyl‐l‐homocysteine are favorable for dimethylation activity. Through NapB5 catalysis, we obtained C‐(di‐)methylated acetylphloroglucinol and flavonoid derivatives. We discovered that NapB5 catalyzes both C‐ and O‐methylation of sterically demanding flavonoids. Here, chemoselectivity was modulated by the geometry of substrate binding through substrate selection or site‐directed mutagenesis. Precise positioning of the acceptor nucleophile toward SAM is required to achieve regio‐ and chemoselectivity despite competing C‐ and O‐nucleophilic sites. Thus, chemoselectivity is context‐dependent, which opens new horizons for the diversification of natural products.

## Graphical abstract

Chemoselectivity is context‐dependent: The SAM‐dependent C‐MTs SfmM2 and NapB5 from streptomycetes catalyze the C‐ and/or O‐dimethylation of aromatic substrates, including l‐tyrosine and flavonoids. C‐methylation activity requires precise substrate recognition and positioning to ensure proximity between the target carbon nucleophile and the methyl group of SAM.

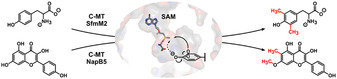

## 1 Introduction

In natural product biosynthesis, *S*‐adenosyl‐l‐methionine (SAM)‐dependent methyltransferases (MTs) play a crucial role in tailoring steps, regulation, and diversification. They catalyze S_N_2‐type methylation reactions and are selective for the nucleophile that acts as acceptor atom [1, 2, 3]. Therefore, SAM‐dependent MTs are typically classified as C‐, O‐, N‐, S‐, or halide MTs depending on their methyl acceptor. The latter are rare examples of known bifunctional or multispecific SAM‐dependent MTs, which accept distinct nucleophiles in the context of their native reaction. They are involved in the biogenesis of methyl halides and methylated sulfur compounds in nature and accept chloride, bromide, iodide, thiol, and the pseudohalide thiocyanate as methyl acceptor [4, 5].

Only few examples of MTs have been reported that are promiscuous in terms of chemoselectivity, catalyzing the methyl transfer to acceptor atoms other than those they have evolved for. For example, the anthranilate N‐MT *Rg*ANMT has been shown to perform O‐methylation (less than 5%) [6] and some catechol and caffeate O‐MTs perform S‐methylation of non‐native thiol substrates (Figure S21C) [7]. Although the general catalytic mechanisms of the S_N_2 methylation reaction are well‐known (acid/base catalysis, proximity and desolvation, or metal‐dependent activation), the mechanisms that determine the chemoselectivity of these enzymes remain poorly understood [7].

Recent studies have demonstrated that substrate protonation states, controlled by conformational dynamics of the enzyme, influence the chemoselectivity of O‐ and N‐MTs (*Pp*CaOMT and *Rg*ANMT, respectively). O‐MTs adopt a closed conformation that facilitates efficient deprotonation of the substrate and prevents phenolate reprotonation, a feature redundant and absent in N‐MTs, as N‐methylation does not require a deprotonation step prior to attack on the methyl donor. Interchanging these properties by site‐directed mutagenesis has led to a reversal of preferences of nucleophile selectivity (Figure S21B) [6].

From a mechanistic perspective, C‐methylation is of particular interest because carbon needs activation by a neighboring functional group to increase the nucleophilicity and enable electrophilic methyl transfer [8, 9]. In the case of aromatic C‐methylation, this activation is often effected by a phenolate, which increases the carbanionic character of the *ortho*‐ and *para*‐carbon. For the aromatic C‐MTs, CouO, NovO, SibL, and PokMT1, it has been postulated that catalytic base‐assisted deprotonation of a hydroxyl group activates the carbon nucleophile [10, 11, 12, 13]. The resonance of the nucleophilic phenolate oxygen and carbanion raises the question of how chemoselective methylation by aromatic C‐MTs is achieved and controlled in nature.

**FIGURE 1:**
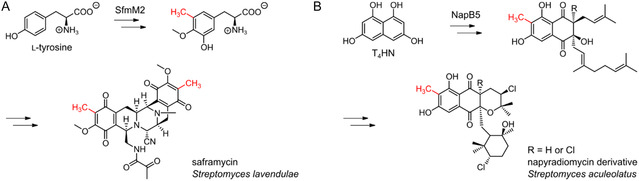
Aromatic C‐methylation in the biosynthesis of saframycin (A) and napyradiomycin (B) catalyzed by the C‐MTs SfmM2 and NapB5, respectively. T_4_HN: 1,3,6,8‐tetrahydroxynaphthalene.

The direct methylation of small molecules presents a significant challenge in organic synthesis, where conventional approaches usually require high catalyst loadings, toxic methylation reagents, and stringent reaction conditions that often lead to overalkylation and limited selectivity. Here, we investigated aspects of C‐ and O‐promiscuity and nucleophile selectivity on the example of two SAM‐dependent C‐MTs that catalyze the regio‐ and chemoselective aromatic monomethylation in the biosynthesis of streptomycete natural products. Covering the shikimate pathway and the polyketide pathway, respectively, the two C‐MTs, SfmM2 and NapB5 (20.3%/37.3% sequence identity/similarity), act on structurally distinct aromatic substrates, i.e., monocyclic phenols and bicyclic polyhydroxynaphthalenes: SfmM2 catalyzes the aromatic C‐methylation of l‐tyrosine in the biosynthesis of saframycin in *Streptomyces lavendulae* (Figure 1A) [14, 15]; NapB5 catalyzes the C‐methylation of an 1,3,6,8‐tetrahydroxynaphthalene (T_4_HN)‐type polyketide synthase (PKS) product in the biosynthesis of napyradiomycins in *Streptomyces aculeolatus* (Figure 1B) [16, 17].

## 2 Results and Discussion

### 2.1 Dimethylation Activity of SfmM2

**FIGURE 2:**
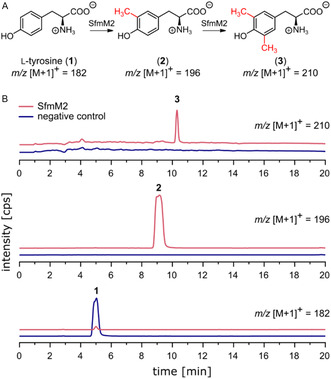
(A) Conversion of l‐tyrosine (**1**) by SfmM2 using an in situ SAM supply cascade with *Ec*MAT and *Ec*MTAN. (B) Extracted ion chromatograms (HPLC‐MS) of SfmM2 activity assay with **1** and the in situ SAM supply cascade. Negative controls were performed without SfmM2.

SfmM2 has previously been described to catalyze the C‐monomethylation of l‐tyrosine (**1**), d‐tyrosine, and l‐DOPA in vitro [18]. We conducted the conversion of **1** using purified His_6_‐tagged SfmM2 and a linear SAM supply cascade, where SAM was biosynthesized in situ from ATP and l‐methionine by methionine adenosyltransferase (*Ec*MAT) and the methylation by‐product‐adenosyl‐l‐homocysteine (SAH) was irreversibly cleaved by methylthioadenosine/SAH nucleosidase (*Ec*MTAN) as a third enzyme in line [19, 20]. High‐performance liquid chromatography–mass spectrometry (HPLC–MS) analysis of this activity assay showed two product peaks with the main product corresponding to the known monomethylation product 3‐methyl‐l‐tyrosine (**2**) [18]. The *m/z* ratio of the second product ([M + 1]^+^ = 210) corresponds to a newly observed dimethylation product (Figure 2). ^13^C NMR experiments of SfmM2 activity assays with **1** and in situ generated labeled [^13^C‐methyl]‐SAM as methyl donor confirmed that both methylations are C‐chemoselective (15.2 and 15.6 ppm, Figure S12) with 3,5‐dimethyl‐l‐tyrosine (**3**) being the dimethylation product. Previous findings demonstrated that MT activity increases with the use of the SAM supply cascade [19], particularly due to SAH removal, as SAM is inherently unstable (prone to nonenzymatic epimerization and degradation) and SAH competitively inhibits many MTs [21, 22, 23]. Applying this cascade in vitro, the O‐MT *Mx*SafC exhibited exceptional catechol O‐dimethylation activity, while other OMTs, such as *Rn*COMT, have not shown such capacity, even in the presence of an excess of methyl equivalents [24]. This system may thus serve as a robust platform for evaluating MT activity at its full catalytic potential, including dimethylation.

The activity of SfmM2 is restricted to phenol (and catechol) amino acids, while compounds without amino acid function or phenolate (desaminotyrosine, tryptamine, 4‐amino‐l‐phenylalanine, and *p*‐coumaric acid) were not converted. SfmM2's peculiarity of high substrate specificity in combination with relaxed stereo preference is shared by homologous and phylogenetically related C‐MTs [18]. Docking experiments of SfmM2 with **1** and comparison with the crystal structure of the tyrosine O‐MT MfnG from the biosynthesis of marformycin (PDB: 7UX8) [25, 26] indicate a dependence of nucleophile selectivity on substrate positioning. In both enzymes, the substrate tyrosine is anchored by interactions with its amino acid moiety. However, compared to MfnG, tyrosine in SfmM2 is rotated by 90°, resulting in the proximity between the methyl donor and the nucleophilic carbon (Figure S2).

### 2.2 Dimethylation Activity of NapB5

**FIGURE 3:**
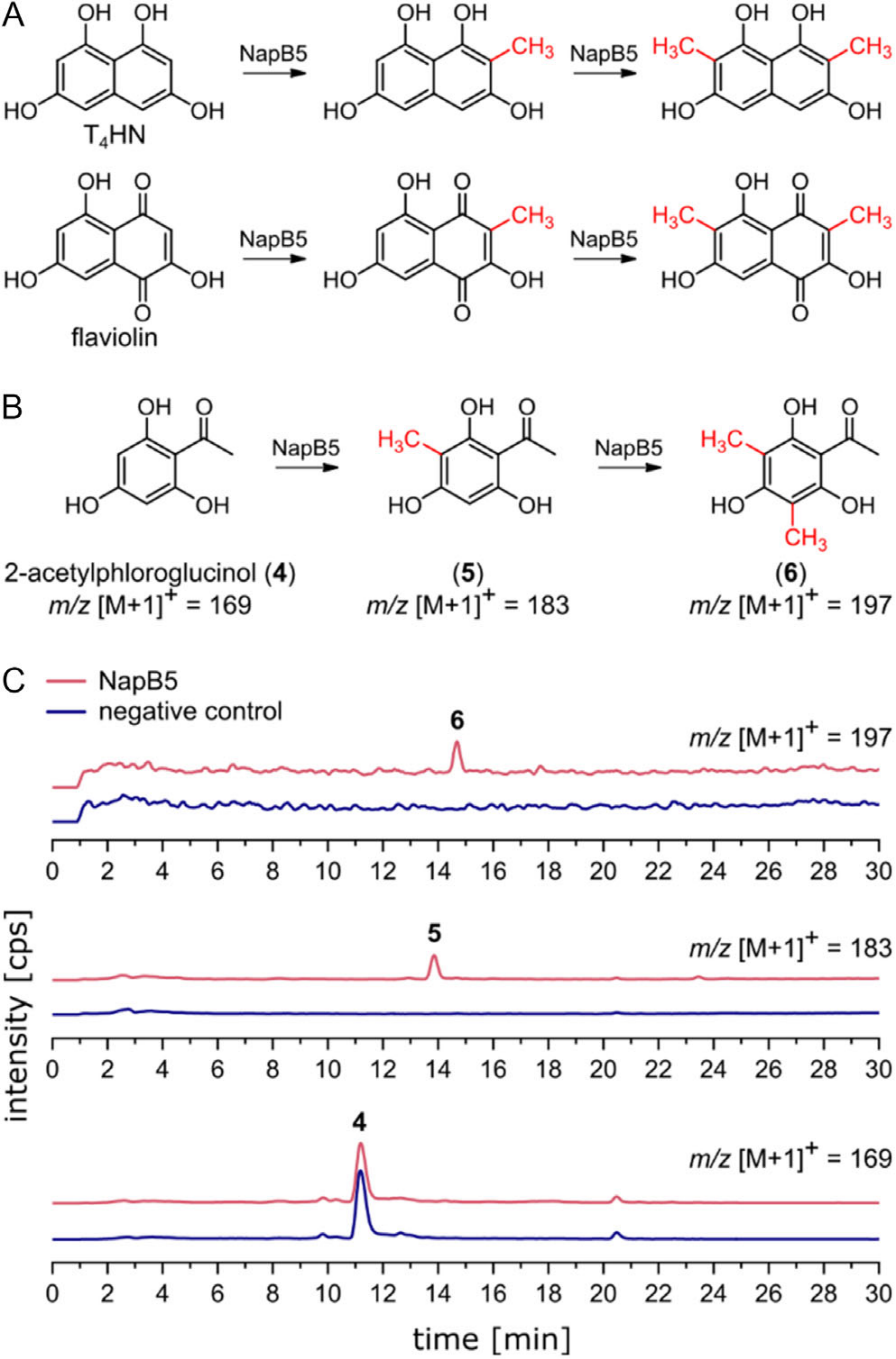
(A) Mono‐ and dimethylation of T_4_HN and flaviolin by NapB5. (B) Conversion of 2‐acetylphloroglucinol (**4**) by NapB5 using an in situ SAM supply cascade with *Ec*MAT and *Ec*MTAN. (C) Extracted ion chromatograms (HPLC‐MS) of NapB5 activity assay with **4** and the in situ SAM supply cascade. Negative controls were performed without NapB5.

Non‐native C‐dimethylation activity is not unique to the active site architecture of SfmM2, as we recently demonstrated in vitro dimethylation activity of NapB5 (even at equimolar SAM concentrations) for the substrate analogs T_4_HN and flaviolin (Figure 3A) [27]. Given the symmetric binding geometry of these substrates, we hypothesized that the dimethylation activity of NapB5 might extend beyond substrates with T_4_HN or naphthoquinone core structures to other symmetric polyhdroxylated aromatic compounds. A substrate screening with 2,7‐dihydroxynaphthalene, 1,3,5‐trihydroxybenzene (phloroglucinol), its derivatives 2,4,6‐trihydroxyacetophenone (2‐acetylphloroglucinol, **4**), 2,4,6‐trihydroxybenzoic acid, and 2,4‐dihydroxy‐6‐methylacetophenone was performed using the linear SAM supply cascade of *Ec*MAT, NapB5, and *Ec*MTAN. Of these compounds, only the symmetric substrate **4** underwent chemoselective mono‐ and dimethylation (Figure 3B,C) yielding 2‐acetyl‐4‐methylphloroglucinol (**5**) and 2‐acetyl‐4,6‐dimethylphloroglucinol (**6**), which was confirmed by ^1^H nuclear magnetic resonance (^1^H NMR) and heteronuclear single quantum coherence (HSQC) experiments (Figures S13 and S14). In comparison, the resorcinol derivative 2,4‐dihydroxy‐6‐methylacetophenone was monomethylated (traces) with no detectable dimethylation product. Neither phloroglucinol nor 2,7‐dihydroxynaphthalene were accepted as substrates (Figures S3 and S6), suggesting that a 2,4‐dihydroxyacetophenone pattern is favorable for substrate binding and thus methylation (Figure S8). Additional substrate symmetry appears to be advantageous for chemoselective C‐dimethylation. The negative charge of 2,4,6‐trihydroxybenzoic acid probably hinders substrate binding.

### 2.3 Promiscuous C‐ and O‐Methylation of Flavonoids

**FIGURE 4:**
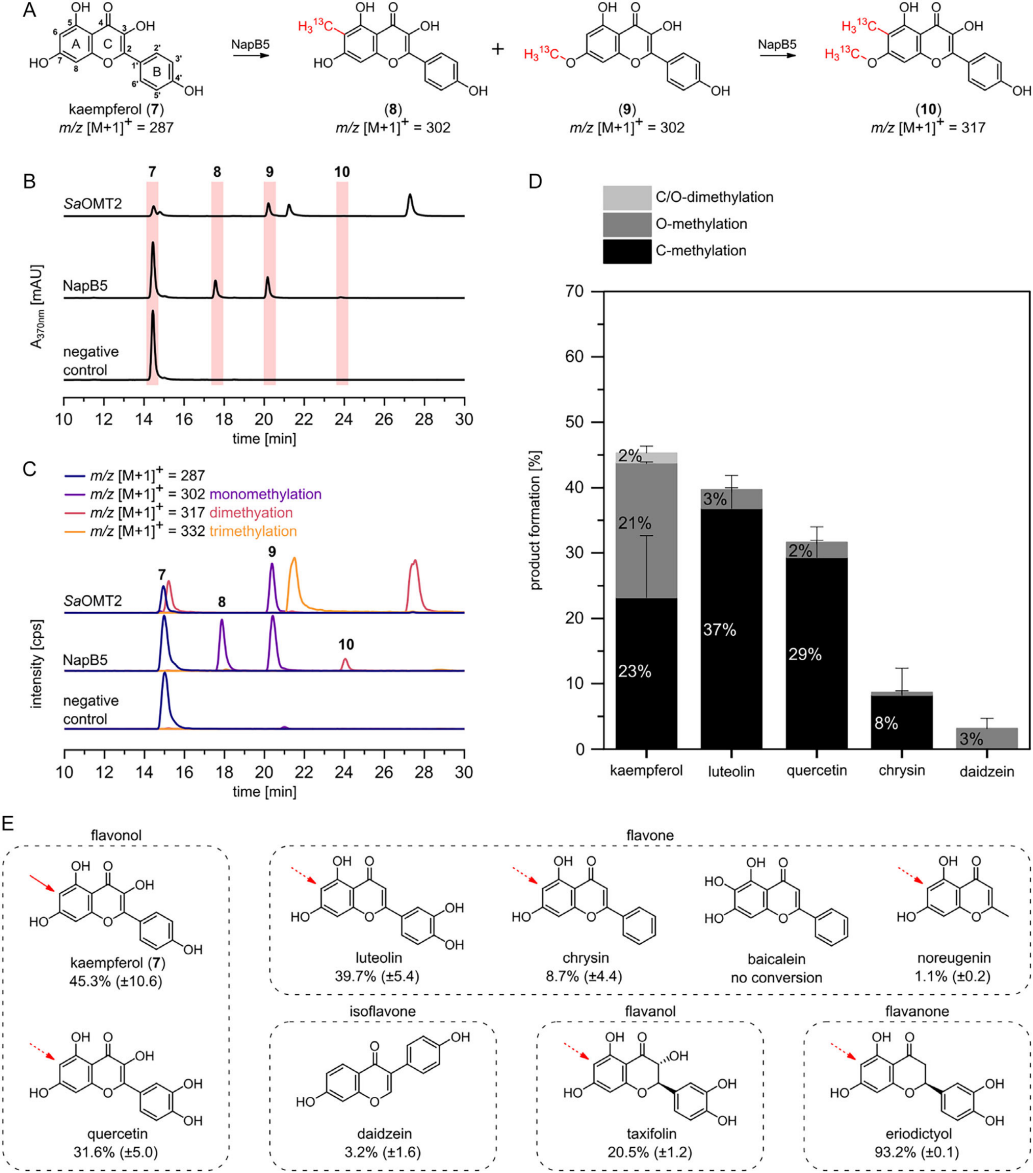
(A) Conversion of kaempferol (**7**) by NapB5 using an in situ [^13^C‐methyl]‐SAM supply cascade with *Ec*MAT and *Ec*MTAN. Negative controls were performed without NapB5. (B and C) HPLC‐DAD and extracted ion chromatograms (HPLC‐MS) of NapB5 and *Sa*OMT2 activity assays with **7** and the in situ [^13^C‐methyl]‐SAM supply cascade. Monomethylated product **9** appears as identical peak in activity assays with the O‐MT *Sa*OMT2. (D) Product formation in NapB5 activity assays with flavonoid substrates and the SAM supply cascade. (E) Product formation (mean [%] ± standard deviation) for conversion of flavonoids by NapB5 calculated based on HPLC‐DAD analysis. Putative C‐methylation sites are marked with red arrows.

Given the resonance of the nucleophilic phenolate oxygen and the observed importance of substrate positioning for the chemoselective (di)methylation activity of SfmM2 and NapB5, we hypothesized that a sterically hindered binding geometry might alter chemoselectivity. To test this, flavonoids serve as promising substrates for NapB5, as they constitute a 2,4‐dihydroxyacetophenone moiety important for substrate binding in combination with a sterically demanding aromatic substituent at position C2 (ring B) that may influence binding in NapB5. Following this approach, we evaluated the enzymatic conversion of flavonols, flavones, isoflavones, flavanols, and flavanones by NapB5 (Figure 4E). HPLC‐DAD and MS analysis of extracted activity assays with the flavon‐3‐ol kaempferol (**7**) and in situ generated [^13^C‐methyl]‐SAM showed the formation of three products: the *m/z* ratio of two products correspond to ^13^C‐labeled monomethylation ([M + 1]^+^ = 302) and the *m/z* ratio of the third product to ^13^C‐labeled dimethylation ([M + 1]^+^ = 317) (Figure 4B,C). Surprisingly, one of the monomethylation product peaks appears identically in activity assays with the O‐MT *Sa*OMT2 from *S. avermitilis*, which was used to generate O‐methylated reference compounds. *Sa*OMT2 has been described as a regiospecific 7‐O‐MT that accepts a broad range of flavones and isoflavones, as well as the coumarin umbelliferone [28, 29]. Hence, our results suggest the C‐ and O‐promiscuous methylation of **7** by NapB5, resulting in 6‐methylkaempferol (**8**) and 7‐*O*‐methylkaempferol (**9**). The formation of both products was confirmed by NMR experiments (Figure S15–17) with the ^13^C‐labeled methyl groups of **8** and **9** appearing as ^1^ *J*_CH_ doublets (128 Hz) at 2.09 and 3.92 ppm for the C6‐methyl group of **8** and the 7‐O‐methyl group of **9**, respectively. The products were verified by comparison with *Sa*OMT2 assays and HSQC correlations (Figure S19). Given the combination of both methylation sites, we assigned the dimethylation product **10** as 6‐methyl‐7‐*O*‐methylkaempferol (Figure 4A). All other flavonoids tested were converted by NapB5, with the exception of the 6‐hydroxyflavone baicalein, in which the putative methylation site at C6 is substituted with a sterically hindering hydroxy group (Figures 4E, S18, and S20).

Based on HPLC‐DAD analysis of NapB5 and *Sa*OMT2 assays, the ratio of C‐ and O‐methylated products was calculated for the NapB5‐catalyzed conversion of kaempferol (**7**), luteolin, quercetin, chrysin, and daidzein (Figure 4D). For **7**, C‐ and O‐methylation occurred almost equimolar. It is likely that the sterically demanding ring B forces the substrate into a binding position where the O‐ and C‐nucleophiles compete for nucleophilic attack on SAM. Docking experiments show that the enzyme's compact binding pocket tightly encloses the substrate (Figure S10).

In comparison, C‐methylation increased substantially for luteolin and quercetin, with no detectable dimethylation. Interestingly, chrysin underwent almost exclusive C‐methylation (≤0.5% O‐methylation), which might be due to a less steric hindrance of ring B compared to **7**, luteolin, and quercetin. Thus, the hydroxylation pattern of ring B appears to influence the binding geometry. Daidzein underwent only O‐methylation (see Figure 4E for total conversions). Its isoflavone structure lacks a 2,4‐dihydroxyacetophenone pattern, resulting in a complete loss of C‐methylation while retaining O‐methylation.

**FIGURE 5:**
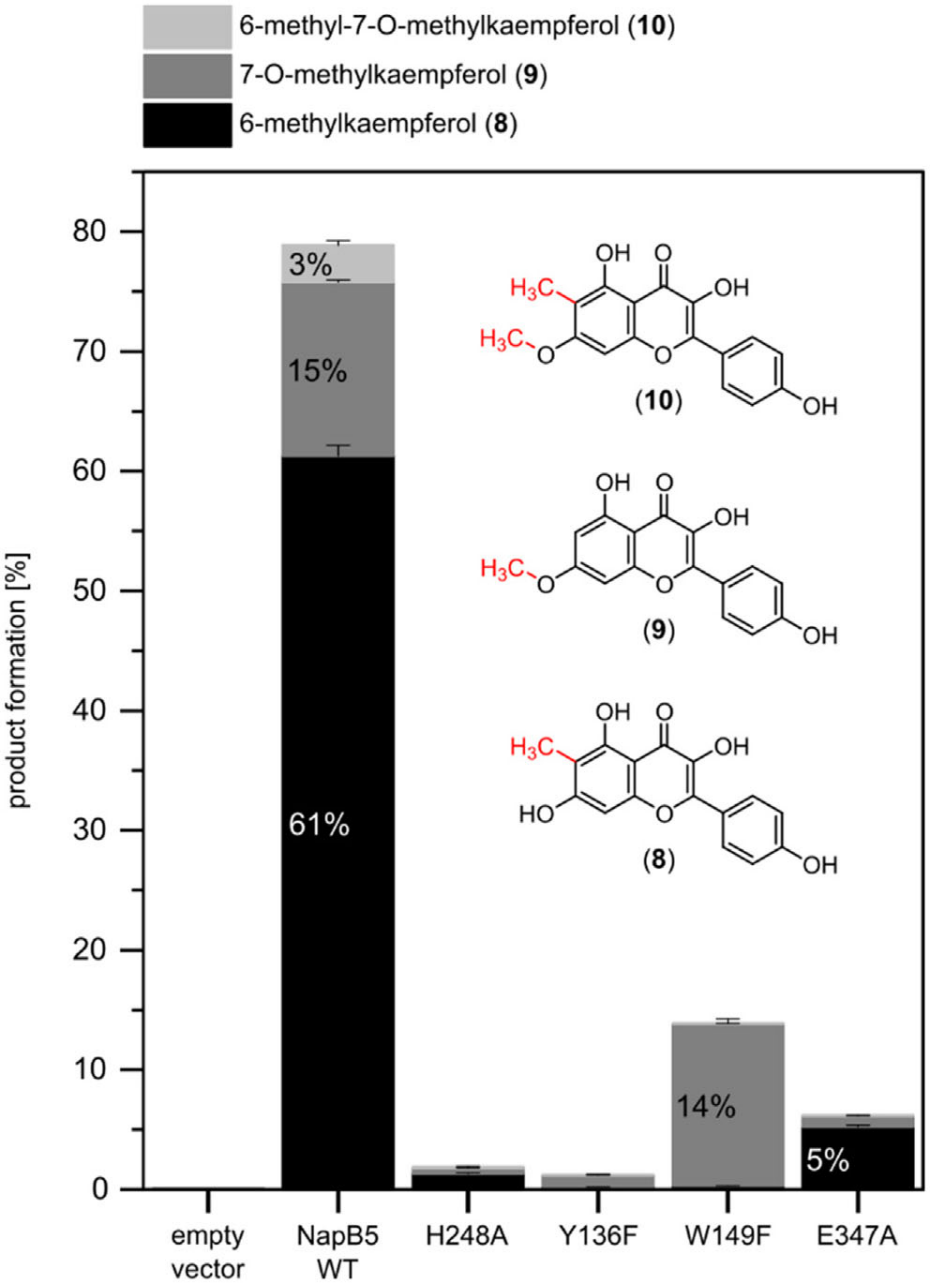
Product formation in lysate activity assays of NapB5 WT and variants with kaempferol (**7**) and in situ SAM supply cascade with *Ec*MAT and *Ec*MTAN. Mean [%] (± standard deviation) was calculated based on HPLC‐DAD analysis at 370 nm.

The substrate screening demonstrates that the selectivity of NapB5 is substrate‐dependent, suggesting that selectivity could also be modulated through point mutations of substrate binding residues. We tested this hypothesis by assaying the NapB5 single variants H248A, Y136F, W149F, and E347A, in comparison to the wild‐type (WT) enzyme, with **7** (Figures 5 and S9) using cell‐free lysate. His‐248 and Tyr‐136 exhibit key substrate‐binding interactions, while Trp‐149 and Glu‐347 sterically constrain the binding pocket [27]. Replacing these positions with hydrophobic and sterically less demanding residues may reduce hydrophilic interactions and influence substrate positioning. The chemoselectivity of **7** methylation can be controlled to a certain extent by the variants W149F and E347A. Both variants exhibited reduced activity compared to the WT, but W149F catalyzed exclusively O‐methylation and E347A retained C‐methylation activity with less than 1% of O‐methylated product. Mutations of the putative catalytic residues Tyr‐136 and His‐248 resulted in almost complete loss of activity. Combined with the previous observation that Glu‐347 and Trp‐149 are of structural importance and not directly involved in the catalytic mechanism [27], these findings suggest that NapB5's selectivity is determined by substrate positioning and proximity between the attacking nucleophile and the methyl group of SAM.

The conversion of **7** by NapB5 WT increased substantially in lysate assays (78.9% ± 1.4) compared to purified enzyme (45.3% ± 10.6). This may arise from crowding effects and enhanced stability of NapB5 in lysate, which could also account for the higher standard deviation of conversion of **7** by purified NapB5. Similar effects have been observed in NapB5 lysate assay with flaviolin [27].

Interestingly, the O‐MT *Sa*OMT2 showed promiscuous O‐methylation activity on flavonoids under the given testing conditions. In addition to the previously published 7‐O‐methylation [28], HPLC‐MS results demonstrated that *Sa*OMT2 catalyzes up to tetramethylation (O‐selective) as observed for quercetin, taxifolin, and eriodictyol conversion (Figure S7). Its (regio‐)promiscuity extends to substrates beyond umbelliferone and flavonoids, including 2‐acetylphloroglucinol (**4**), 2,4‐dihydroxy‐6‐methylacetophenone, 2,4,6‐trihydroxybenzoic acid, 2,7‐dihydroxynaphthalene, T_4_HN, and flaviolin (Figures S3–S6).

Compared to ubiquitous O‐methyl derivatization, C‐methylation of flavonoids is rarely found in natural products. Especially, microbial C‐methylated flavonols have not yet been reported [30]. In the domain of plants, several C‐methylated flavonoids and chalcones have been isolated, e.g., from *Cleistocalyx* spp. [31, 32], *Pisonia grandis* [33], *Pentarhizidium orientale* [34, 35], *Myrica gale* [36], *Cetrus deodara* [37], and from numerous Myrtaceae species (Figure S11C) [38, 39, 40, 41, 42, 43]. The latter family is also rich in C‐methylated acylphloroglucinol derivatives, which are structurally and biosynthetically related to flavonoids (Figure S11) [44, 45]. These compounds are of particular interest due to their uncommon structure, their bioactivity, and pharmacological properties. It was shown that C‐methylated flavonoids, chalcones, and acylphloroglucinols exhibit antimicrobial, anti‐inflammatory, antiviral, antioxidant, and radical scavenging activity [31, 36, 46, 47, 48, 49]. For example, the ‘magic methyl effect’ of the C8‐methylation in eucalyptin was elaborated recently, causing significant alterations in cytotoxic and antibiofilm activities [46]. Additionally, these compounds are of significant interest in the context of natural product diversification, as regioselective C‐monomethylation, C‐dimethylation, including geminal dimethylation, dimerization, and O‐methylation of flavonoids and acylphloroglucinols are found in plant extracts. Notable examples of C‐dimethylated and/or dimerized compounds are flavesone [48], saroaspidin A [49], and baeckenone C [42]. The biosynthetic pathways of flavonoids and acylphloroglucinols are related, with C‐methylation proposed to occur either as post‐PKS tailoring modification on the aromatic core structure [32, 45], and/or during PKS assembly through incorporation of methylmalonyl‐CoA as a building block (Figure S11A,B) [38, 50]. Recently, the first chalcone C‐MT was discovered [51]. RdCMT (15.5%/29.1% sequence identity/similarity with NapB5) from *Rhododendron dauricum* catalyzes the C‐dimethylation of naringenin chalcone, leading to the biosynthesis of farrerol. It is proposed that an His–Glu dyad mediates the initial deprotonation of the chalcone, and the enzyme's C‐methylation selectivity is determined through the binding conformation of the substrate (Figure S21B). The identification and characterization of homologous C‐MTs involved in the biosynthesis of related flavonoids and acylphloroglucinols remain elusive [52]. NapB5, which accepts acetylphloroglucinol and diverse flavonoids as substrates, may thus offer valuable insights into the aromatic C‐(di)methylation of these compounds.

The unprecedented O‐methylation activity of WT NapB5 shows that C‐MTs are mechanistically not restricted to C‐methylation. It is likely that C‐MTs were subjected to stronger evolutionary pressure due to the weaker electronegativity of the carbon nucleophile [53]. Activation of the carbon by hydroxyl deprotonation simultaneously enhances the nucleophilicity of the oxygen. An unspecific binding geometry, as in the case of NapB5 with non‐native and sterically demanding flavonoids, leads to the concurrent competition of C‐ and O‐nucleophile. Thus, the precise substrate binding for positioning the nucleophile near the methyl donor is ultimately crucial for chemoselective C‐methylation. Consequently, nucleophile promiscuity of C‐MTs is a rare phenomenon compared to MTs acting on chemically similar and reactive heteroatoms or (pseudo‐)halide nucleophiles. A notable exception is the biosynthesis of nocardioazines from actinomycetes, where the bifunctional NozMT performs sequential N‐ and C‐methylation (Figure S21A) [54].

**FIGURE 6:**
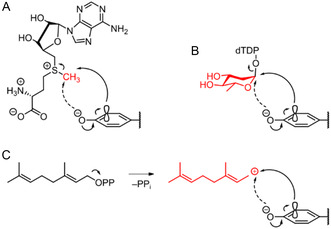
Comparison of aromatic nucleophilic substitution reactions catalyzed by methyl‐, glycosyl‐, and prenyltransferases. (A) C‐ versus O‐methylation of phenolic substrates with SAM as electrophilic methyl donor. (B) C‐ versus O‐ glycosylation of phenolic substrates with dTDP‐olivose as exemplified electrophilic nucleotide diphosphate sugar donor. (C) C‐ versus O‐prenylation of phenolic substrates with geranyl diphosphate as electrophilic prenyl donor.

The dependence of nucleophile positioning and chemoselectivity of an electrophilic aromatic substitution also applies to glycosyltransferases and prenyltransferases (Figure 6). Bechthold's group showed that the urdamycin glycosyltransferase UrdGT2 catalyzes the formation of both C–C and C–O glycosidic bonds [55]. Similar to aromatic methylation, O‐ and C‐glycosylation depends on the positioning of the hydroxyl group or the *ortho*‐carbon of the aromatic substrate relative to the anomeric carbon of the glycosyl donor [56]. Analogously, it has been proposed that the nucleophile selectivity of aromatic C‐prenyltransferases depends on correct positioning of the substrate [57, 58, 59]. Notably, several C‐prenyltransferases that catalyze the tailoring of the naphthoquinone core structure in T_4_HN‐derived meroterpenoid pathways exhibit a recurrent pattern of activity analogous to NapB5, as indicated by their promiscuity for both C‐mono‐ and di‐prenylation of simplified substrates [60] as well as simultaneous C‐ and O‐prenylation of non‐native flavonoids [57].

## 3 Conclusion

**FIGURE 7:**
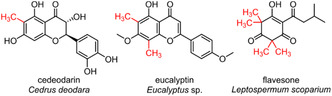
Exemplary C‐methylated acylphloroglucinol and flavonoid natural products.

We demonstrated the unprecedented dimethylation activity of NapB5 and SfmM2 in vitro. In situ SAM supply and SAH removal help to assess MT activities at their full capacity with degrees of methylation, up to tetramethylation in the case of the promiscuous O‐MT *Sa*OMT2. The activity of SfmM2 is restricted to aromatic amino acids and performs chemoselective C‐mono‐ and C‐dimethylation of its native substrate l‐tyrosine (**1**). The relaxed stereo preference of SfmM2 and the symmetry of the substrate allow precise positioning, thereby preserving chemoselectivity of the second methylation. The previously reported NapB5 C‐dimethylation activity toward T_4_HN and flaviolin was extended to the symmetric non‐native substrate 2‐acetylphloroglucinol (**4**), suggesting that a 2,4‐dihydroxyacetophenone pattern combined with symmetric binding geometry is favorable for C‐dimethylation by NapB5. Mechanistically, C‐MTs can exhibit nucleophile promiscuity depending on the positioning of the acceptor atom in the vicinity of SAM. This was proven by NapB5, which catalyzes the C‐ and/or O‐methylation of various flavonoids, including the dimethylation of kaempferol (**7**) yielding 6‐methylkaempferol (**8**), 7‐O‐methylkaempferol (**9**), and 6‐methyl‐7‐*O*‐methylkaempferol (**10**). The enzymatic access to C‐methylated acylphloroglucinols and flavonoids provides a starting point to investigate the role of C‐MTs in the biosynthetic pathways and diversification of interesting natural products, such as the dearomatized β‐triketone flavesone or the C6‐methylflavanol cedeodarin (Figure 7 and S11C). In addition, flavonoid substrate screening of NapB5 WT and variants, together with docking studies of SfmM2 and NapB5 provide valuable insights into the control of chemoselectivity by aromatic C‐MTs. Thus, C‐MTs have evolved to achieve precise nucleophile positioning of their native substrates toward SAM and to accomplish regio‐ and chemoselective activity despite competing C‐ and O‐nucleophilic sites.

Finally, these results illustrate the limitations of enzyme classification and challenge loosely used terms such as ‘chemoselectivity’ in the context of catalytic activity and terminology, i.e., C‐ versus O‐MT. While chemoselectivity typically refers to the selective reactivity of a functional group in the presence of others, the observed nucleophile selectivity of C‐MTs is not determined solely by the relative nucleophilicity of the functional groups. Instead, it ultimately depends on the positioning of the substrate. From a mechanistic point of view, it could therefore be argued that the C6‐ versus 7‐O‐methylation of **7** by NapB5 can be described as regioselectivity rather than chemoselectivity. The present work shows that selectivity is context‐dependent and is influenced by factors such as the choice of substrate (native vs. non‐native), catalytic environment (in vitro vs. biosynthesis; cell‐free lysate vs. purified enzyme), and allosteric (e.g., substrate and/or product inhibition), or competitive regulation (e.g., MT inhibition by SAH). In synthetic organic chemistry, a streamlined approach usually involves the development of highly selective catalysts. Beyond this linear perspective and the ideal of a “perfectly” selective catalyst, enzymatic selectivity can be understood as a conditional property: It arises from the intrinsic structural and conformational flexibility and plasticity of enzymes [61, 62, 63], and is complemented by context‐dependence (and the interdependence of these factors). This synergy opens a dynamic and seamless spectrum of selectivity that represents a fundamental evolutionary adaptive feature and new horizons for diversification.

The need for an open approach to the evaluation of enzyme selectivity and unconventional catalytic activities extends beyond the enzyme class of MTs. For instance, the exceptionally wide substrate scope of d‐fructose‐6‐phosphate aldolase has been shown, challenging conventional paradigms on the narrow nucleophile specificity of aldolases [64]. Additionally, unspecific peroxygenases have been shown to catalyze C–C bond formation, revealing their unexpected role in oxidative phenol coupling during the biosynthesis of natural products [65]. Another striking example is the recently discussed catalytic versatility of NAD(P)H‐dependent oxidoreductases, which exert multiple native reductase activities at different C=X bonds (with X being C, N, or O) exceeding the functional scope implied by the nomenclature and substrate‐associated classification [66].

In summary, promiscuity and relaxed substrate selectivity are not exceptions, but inherent and evolved features of enzymes, serving as a foundation for further evolutionary adaptations due to physicochemical constraints or environmental pressures [62, 67]. Within this framework, chemoselectivity emerges as a conditional rather than an intrinsic property, which by its nature is inevitably perfectly imperfect.

## Supporting Information

Additional supporting information can be found online in the Supporting Information section.

## Funding

The work was supported by the Deutsche Forschungsgemeinschaft (FOR 5596 and 510974120) and Chinese Scholarship Council (202204910052).

## Conflicts of Interest

The authors declare no conflicts of interest.

## Supporting information

Supplementary Material

## Data Availability

The data that support the findings of this study are openly available in zenodo.org at https://doi.org/10.5281/zenodo.17160368, reference number 17160368.
