# Supplementary material for: Context‐Dependent Chemoselectivity of Aromatic C‐Methyltransferases
Source: Chembiochem. 2026 Apr 24;27(8):e70294. doi: 10.1002/cbic.70294 (PMC13109631; doi:10.1002/cbic.70294)
Supplement: Supplementary file 1 — Supplementary Material [file CBIC-27-e70294-s001.pdf]

Supporting Information

Context-Dependent Chemoselectivity of Aromatic C-Methyltransferases

Juliane Breiltgens,[a] Ziruo Zou,[a] Sascha Ferlaino,[a] Jennifer N. Andexer,[a] and Michael Müller*[a]

[a] Institute of Pharmaceutical Sciences, University of Freiburg, Albertstrasse 25, 79104 Freiburg, Germany,

michael.mueller@pharmazie.uni-freiburg.de

1. Experimental procedures 4

1.1. Analytical methods 4

1.2. Plasmid construction and site-directed mutagenesis 4

1.3. Protein production and purification 4

1.4. In vitro activity assays 5

1.5. Bioinformatic Analysis 5

2. Sequences 6

3. ^{1}H NMR chemical shifts of 4, 5, 7, 8, and 9 7

4. Supplementary Tables 8

5. Supplementary Figures 10

6. Supplementary References 26

Figure S1: SDS-PAGE of His_6-tagged MTs and bovine serum albumin (BSA) standard (1 mg·mL^{-1}) after Ni-NTA purification. Ladder: NEB Color Prestained Protein Standard. X: enzyme not used in this study. 1: SfmM2 (43.8 kDa). 2: NapB5 (43.2 kDa). 3: *Sa*OMT2 (41.3 kDa). 4: BSA standard (66.5 kDa)........ 10

Figure S2: Active site of superimposed tyrosine O-MT MfnG (magenta; co-crystallized with SAH and soaked with L-tyrosine; PDB: 7UX8) and tyrosine C-MT SfmM2 (green; AlphaFold2; docked with L-tyrosine). The respective acceptor nucleophiles are located near the sulfur of SAH. 10

Figure S3: HPLC-DAD chromatograms of NapB5 and *Sa*OMT2 activity assay with 2,7-dihydroxynaphthalene using an in situ *S*-adenosyl-L-methionine (SAM) supply cascade with *Ec*MAT and *Ec*MTAN. No conversion by NapB5 was observed. Putative O-methylated products of *Sa*OMT2 are marked with an asterisk. 11

Figure S4: HPLC-DAD chromatograms of NapB5 and *Sa*OMT2 activity assay with 1,3,6,8-tetrahydroxynaphthalene (T_4HN) or flaviolin using an in situ *S*-adenosyl-L-methionine (SAM) supply cascade with *Ec*MAT and *Ec*MTAN. Putative O-methylated products of *Sa*OMT2 are marked with an asterisk and show distinct retention times compared to the C-methylated products of NapB5 (3-methylflaviolin and 3,6-dimethylflaviolin), which have been characterized in our previous study.[1] ... 11

Figure S5: Extracted ion chromatograms (HPLC-MS) of NapB5 and *Sa*OMT2 activity assays with: **A**) 2-acetylphloroglucinol (**4,** m/z $[M+1]^+$ = 169) using an in situ *S*-adenosyl-L-methionine (SAM) supply cascade with *Ec*MAT and *Ec*MTAN. The m/z values of substrate ($[M+1]^+$), mono- (+14), and dimethylation (+28) are shown; **B**) 2,4-dihydroxy-6-methylacetophenone (m/z $[M+1]^+$ = 167) using an in situ [^{13}C-methyl]-SAM supply cascade with *Ec*MAT and *Ec*MTAN. The m/z values of substrate ($[M+1]^+$), mono- (+15), and dimethylation (+30) are shown. 12
Figure S6: Extracted ion chromatograms (HPLC-MS) of NapB5 activity assays with: **A**) phloroglucinol (m/z $[M+1]^+$ = 127); **B**) 2,4,6-trihydroxybenzoic acid (m/z $[M+1]^+$ = 171) using an in situ [^{13}C-methyl]-SAM supply cascade with *Ec*MAT and *Ec*MTAN. The m/z values of substrate ($[M+1]^+$), mono- (+15), and dimethylation (+30) are shown. 12
Figure S7: Extracted ion chromatograms (HPLC-MS) of NapB5 and *Sa*OMT2 activity assays with flavonoids using an in situ [^{13}C-methyl]-*S*-adenosyl-L-methionine (SAM) supply cascade with *Ec*MAT and *Ec*MTAN. The m/z values of the substrate ($[M+1]^+$) and mono-/di-/tri-/tetramethylation are shown. O-methylated products by NapB5 appear as identical peaks in activity assays with the O-MT *Sa*OMT2. 14
Figure S8: Compounds tested in NapB5 activity assays. The 2,4-dihydroxyacetophenone pattern favorable for substrate binding is highlighted. 14
Figure S9: HPLC-DAD chromatograms (370 nm) of *Sa*OMT2, NapB5 wildtype (WT) and NapB5 variants activity assays with kaempferol (**7**) using an in situ *S*-adenosyl-L-methionine (SAM) supply cascade with *Ec*MAT and *Ec*MTAN. NapB5 WT and NapB5 variants assays were performed with cell-free lysate of *E. coli* BL21Gold (DE3) cells transformed with the respective constructs. Cell-free lysate of *E. coli* BL21Gold (DE3) cells transformed with the empty vector pET28a was used for the negative control. 14
Figure S10: Docking of kaempferol (**7**) and *S*-adenosyl-L-methionine (SAM) into an AlphaFold2 model of NapB5. 15
Figure S11: **A**) Proposed biosynthetic pathways of C-methylated acylphloroglucinols in plants. **B**) Proposed biosynthetic pathways of C-methylated chalcones and flavonoids in plants. **C**) C-methylated acylphloroglucinol, chalcone, and flavonoid natural products. 16
Figure S12: ^{13}C NMR spectra (D_2O) of SfmM2 activity assays with L-tyrosine (**1**) and negative control without enzyme. The signals of the ^{13}C-labeled methyl groups of [^{13}C-methyl]-L-methionine, [^{13}C-methyl]-SAM, **2**, and **3** are assigned. 17
Figure S13: ^{1}H NMR spectrum (acetone-d_6) of extracted NapB5 assays with 2-acetylphloroglucinol (**4**). The chemical shifts of **4** and **5** are listed on page 7. 18
Figure S14: HSQC spectrum (acetone-d_6) of extracted NapB5 assays with 2-acetylphloroglucinol (**4**). The signals of the ^{13}C-labeled methyl groups of **5** and **6** correlate with the $^1J_{CH}$ doublets of the ^{1}H spectrum. 19
Figure S15: Comparison of ^{1}H NMR spectra (acetone-d_6) of extracted NapB5 and SaOMT2 activity assays with kaempferol (**7**). The protons of the ^{13}C-labeled methyl groups appear as doublet with a heteronuclear coupling ($^1J_{CH}$) constant of 128 Hz. The signals of the aromatic protons of ring B appear as an AA'BB' system. The chemical shifts of **7**, **8** and **9** are listed on page 7. 20
Figure S16: ^{1}H NMR spectrum (acetone-d_6) of extracted NapB5 assays with kaempferol (**7**). The chemical shifts of **7**, **8** and **9** are assigned in Figure S15 and listed on page 7. 21
Figure S17: ^{1}H NMR spectrum (acetone-d_6) of extracted *Sa*OMT2 assays with kaempferol (**7**) yielding **9** and other O-methylated products. The chemical shifts of **7** and **9** are assigned in Figure S15 and listed on page 7. 22
Figure S18: ^{13}C NMR spectra (acetone-d_6) of extracted NapB5 and *Sa*OMT2 activity assays with flavonoids. Due to low substrate concentrations, only ^{13}C-labeled methyl signals are visible. Signals of O-methylation appear at 55–57 ppm and signals of C-methylation at 7.5 ppm. For samples of luteolin with

NapB5, quercetin with NapB5, daidzein with NapB5, and chrysin with NapB5 acetone-d_6 with 0.03% TSM was used as solvent. The conversion of daidzein and chrysin by NapB5 is too low to be detectable in the ^{13}C NMR spectra, but the corresponding signals are visible in the HSQC spectra (Figure S20 C and D)..... 23
Figure S19: HSQC spectra (acetone-d_6) of: **A**) extracted NapB5 assays with kaempferol (**7**); **B**) extracted *Sa*OMT2 assays with **7** 23
Figure S20: HSQC spectra (acetone-d_6 with 0.03% TSM) of extracted NapB5 assays with **A**) quercetin; **B**) luteolin; **C**) chrysin; **D**) daidzein.. 24
Figure S21: Native and modulated nucleophile selectivity of MTs. **A**) Native bifunctional MT NozMT.[8] **B**–**D**) Induced modulation of chemoselectivity by protein engineering of *Rg*ANMT, *Pp*CaOMT,[9] and *Rd*CMT[10] (**B**), or by substrate choice for *Rn*COMT[11] (**C**) and NapB5 (**D**). 25

1. Experimental procedures

1.1. Analytical methods

High-performance liquid chromatography (HPLC) analyses were performed on a 1260 Infinity II LC system (Agilent Technologies) with photodiode array detector (DAD). An Agilent Poroshell 120 (RP-C18), 4 x 150 mm, 4.6 µm with a corresponding precolumn from Agilent Technologies served as stationary phase; the injection volume was 5 µL. The same column and gradient were used for HPLC-MS analysis on an Agilent HP 1100 system with an ESI-QTRAP 4500 mass detector (AB Sciex) operated in positive ionization mode.

Acetophenone derivatives and flavonoids: The mobile phase with a flow rate of 0.225 $mL \cdot min^{-1}$ was composed of 60% H_2O with 0.1% formic acid (A) and 40% ACN (B). Gradient: 0–0.5 min 60% A/ 40% B, 0.5–21.5 min ramp to 15% A/ 85% B, 21.5–26 min ramp to 2% A/ 98% B with a flow rate of 0.300 $mL \cdot min^{-1}$, 26–32 min return to 60% A/ 40% B with flow rate of 0.225 $mL \cdot min^{-1}$, hold 32–35 min. DAD see Table S3.

L-Tyrosine (**1**): The mobile phase with a flow rate of 0.5 $mL \cdot min^{-1}$ was composed of 95% H_2O with 0.1% formic acid (A) and 5% acetonitrile (B). Gradient: 0–3 min 95% A/ 5% B, 3–17 min ramp to 5% A/ 95% B, hold 17–20 min, 20–30 min return to 95% A/ 5% B. DAD at 276 nm.

Assays with flaviolin and T_4HN (Figure S4) were analyzed as described elsewhere.[1]

Nuclear Magnetic Resonance Spectroscopy (NMR) spectra were recorded on an Avance III HD spectrometer (Bruker, Rheinstetten, Germany) at 24 °C using 400 MHz for ^1H NMR spectra and 100.6 MHz for ^{13}C NMR. Chemical shifts (δ) are reported in ppm using the solvent as the internal standard.

1.2. Plasmid construction and site-directed mutagenesis

The plasmid construction of pET28a::*napB5* and site-directed mutagenesis based on this construct were described elsewhere.[1]
The genes *sfmM2* from *S. lavendulae* and *saomt2* from *S. avermitilis* were purchased from Thermo Fisher Scientific as codon-optimized genes for *E. coli*. The genes were amplified using the oligonucleotides listed in Table S2. All reactions were performed in 20 µL total volume using Phusion® High-Fidelity PCR Master Mix with HF Buffer (NEB) following the manufacturer's instructions. PCR parameters are given in Table S1. The resulting amplicon was ligated to an EcoRI-linearized pET28a vector using the In-Fusion® Snap Assembly Master Mix (Takara Bio Europe) to yield expression plasmid pET28a::*sfmM2* and pET28a::*saomt2*, respectively. The constructs were sequenced by Eurofins Genomics Germany GmbH (Ebersberg, Germany) using Sanger sequencing.

1.3. Protein production and purification

The production and purification of His_6-NapB5, His_6-NapB5 variant lysate, His_6-SfmM2, and His_6-*Sa*OMT2 were performed analogously as described elsewhere.[1]
The production and purification of methionine adenosyltransferase (*Ec*MAT) and methylthioadenosine/SAH nucleosidase (*Ec*MTAN) used in the linear SAM supply cascade were performed as described elsewhere.[2,3]

1.4. In vitro activity assays

NapB5 and *Sa*OMT2 activity assays were carried out in triplicates in 100 µL reactions. SAM or [^{13}C-methyl]-SAM was generated in situ from ATP and L-methionine or [^{13}C-methyl]-labeled L-methionine, respectively, by *Ec*MAT. *Ec*MTAN was used to irreversibly cleave the SAH yielding *S*-ribosyl-L-homocysteine and adenine.[2,3] Reactions with purified His_6-NapB5 or His_6-*Sa*OMT2 contained 50 mM Tris-HCl buffer pH 7.5, 0.5–1 mM substrate (dissolved in methanol), 50 mM $MgCl_2$, 20 mM KCl, 3 mM ATP, 3 mM L-methionine, 10 µM *Ec*MAT, 3 µM *Ec*MTAN, and 30 µM MT. Reactions were incubated 20 h at 30 °C with shaking (450 rpm), quenched with 10 µL 1 M HCl, and extracted 3× with ethyl acetate. The organic phase was evaporated, and residues were dissolved in 100 µL methanol for HPLC analysis. Negative controls were performed without the addition of MT. For NMR analysis, 50 reaction extracts were pooled, dried, and dissolved in acetone-d_6. NapB5 wildtype (WT) and NapB5 lysate assays were performed with 50 µL cell-free lysate instead of purified enzyme. Negative controls contained cell-free lysate of *E. coli* BL21Gold (DE3) cells transformed with the empty vector pET28a. SDS-PAGE analysis confirmed that the lysate concentrations of all enzyme variants were comparable, ensuring that the observed differences in activity were due to the introduced mutations and not to variations in the amount of enzyme.[1]

SfmM2 activity assays were performed analogously, but with 2 mM substrate [L-tyrosine (**1**) in 30 mM NaOH] and 50 mM Tris-HCl buffer pH 8. After incubation, the reactions were quenched with 10 µL 1 M HCl, centrifuged (14000 rcf, 5 min), filtered and the aqueous solution was directly injected into the HPLC. Negative controls were performed without the addition of MT. Assays for NMR experiments were scaled up to 500 µL. After incubation, the aqueous solution was measured after centrifugation and addition of 50 µL D_2O.

1.5. Bioinformatic Analysis

Pairwise sequence alignments of protein sequences were performed using EMBOSS Needle (EBLOSSUM62).[4] The SfmM2 and NapB5 structure models were generated using ColabFold v1.5.5: AlphaFold2.[5,6] Docking studies were performed with AutoDock4 using the graphical user interface AutoDockTools.[7] Preparation of macromolecules and ligands (adding polar hydrogens and Kollman charges), grid parameters (50 x-points, 50 y-points, 50 z-points), and genetic algorithm parameters were executed keeping the default settings of AutoDockTools (adjusting the number of requested GA dockings = 50 runs and the population size = 300). Dockings were executed using the Lamarckian genetic algorithm, with a maximum of 2500000 energy evaluations. The docking poses shown (Figures S2 and S10) belong to the conformational cluster with the lowest energy state.

2. Sequences

Protein sequence of His$_6$-NapB5 (43.2 kDa)

*MGSSHHHHHHSSGLVPRGSHMASMTGGQQMGRGSEL*LTPEATFTRFREYMVGPSRFMSLLSCFELGVIDNLRDTPGMTAAELGDAVGAKPDAV
EQLLLLLVKESFLAYDEDTGGYSLDALADIAEDDLRQVLARMEMIKVVTLRQLFYLTDSVRTGTTVGLNKLYGHEGNLYDALAEHKDLREPWARLMN
GETAHIDPWFFDNIDVPPGSNVLDLAGNTGLGAIHTCKLKASPGLRVTTFDLPEKEEECLANFRSHGVEEHCSFIGGDVFEEVPKGFDVVLIKHFLPMF
DKSDVFKILEGVNRSMDVGGQVHLLVPVFPENIKDSDNYTVDFFPSFFIGCAMGQGGAQKMSTWQKWLEECGFTVTKAIAEDPADMLPHALPVE
AVLSATKRAG

Nucleotide sequence of *napB5* (codon-optimized for *E. coli*)

CTGACACCGGAAGCAACCTTTACACGTTTTCGTGAATATATGGTTGGTCCGAGCCGTTTTATGAGCCTGCTGAGCTGTTTTGAACTGGGTGTTA
TTGATAATCTGCGTGATACACCGGGTATGACCGCAGCAGAACTGGGAGATGCAGTTGGTGCAAAACCGGATGCCGTTGAACAACTGCTGCTG
CTGTTAGTTAAAGAAAGTTTTCTGGCCTATGATGAAGATACCGGTGGTTATAGCCTGGATGCACTGGCAGATATTGCAGAAGATGATCTGCGT
CAGGTTCTGGCACGTATGGAAATGATTAAAGTTGTTACCCTGCGCCAGCTGTTTTATCTGACCGATAGCGTTCGTACCGGCACCACCGTTGGTC
TGAATAAACTGTATGGTCATGAAGGCAATCTGTATGATGCCCTGGCCGAACATAAAGATCTGCGCGAACCGTGGGCACGTCTGATGAATGGT
GAAACCGCACATATTGATCCGTGGTTTTTCGATAATATTGATGTTCCGCCTGGTAGCAATGTTCTGGATCTGGCAGGTAATACCGGTCTGGGTG
CAATTCATACCTGTAAACTGAAAGCAAGTCCGGGTCTGCGTGTTACCACCTTTGATCTGCCGGAAAAAGAAGAAGAATGTCTGGCAAATTTTC
GTAGCCATGGTGTTGAAGAACACTGCAGCTTTATTGGTGGTGATGTTTTTGAAGAAGTGCCGAAAGGTTTTGATGTGGTGCTGATTAAACATT
TCCTGCCGATGTTTGATAAAAGCGACGTGTTTAAAATCCTGGAAGGTGTTAATCGTAGTATGGATGTTGGTGGTCAGGTTCATCTGCTGGTTCC
GGTTTTTCCTGAAAACATTAAAGATAGCGATAACTATACCGTGGATTTCTTTCCGAGCTTTTTCATTGGTTGTGCAATGGGTCAAGGTGGTGCA
CAGAAAATGAGCACCTGGCAGAAATGGCTGGAAGAATGTGGTTTTACCGTTACCAAAGCAATTGCCGAAGATCCTGCAGATATGCTGCCGCA
TGCACTGCCGGTTGAAGCAGTTCTGAGCGCAACCAAACGTGCAGGTTAA

Protein sequence of His$_6$-SfmM2 (43.8 kDa)

*MGSSHHHHHHSSGLVPRGSHMASMTGGQQMGRGSEL*MTISLENTTVGQNPAGGPPTGKAPLDMEGLAWILFGASAFQYLNAACELNLFELLEN
KPGLTKPQIGAELGLADRANDILLLGATATGMLTVEDGRYQLATVLAELLKTDDWQRFKDTVGFEQYVCYEGQIDFTESLRSNSNVGLRRVRGSGRD
LYHRLHENPQMEQAFYKYMRSWSELANQHLVEVLDLSGTSKLLDCGGGDAVNSIALAQANPHIEAGILEIPPTAPLTEKKIAEAGLSDRITVKPGDM
HTDEFPTGYDTVMFAHQLVIWTPEENTALLRKAYNALPEGGRVIIFNSMSNDEGDGPVVAALDSVYFAALPAEGGMIYSWATYEESLTKAGFNPET
FQRIDFPGWTPHGVIIATK

Nucleotide sequence of *sfmM2* (codon-optimized for *E. coli*)

ATGACCATTAGCCTGGAAAATACCACCGTTGGTCAGAATCCGGCAGGCGGTCCTCCGACCGGTAAAGCACCGCTGGATATGGAAGGTCTGGC
ATGGATTCTGTTTGGTGCAAGCGCATTTCAGTATCTGAATGCAGCATGTGAACTGAACCTGTTTGAACTGCTGGAAAACAAACCGGGTCTGAC
CAAACCGCAGATTGGTGCAGAACTGGGTTTAGCAGATCGTGCAAATGATATTCTGCTGCTGGGTGCAACCGCAACCGGTATGCTGACCGTTG
AAGATGGTCGTTATCAGCTGGCAACCGTTCTGGCAGAGCTGCTGAAAACCGATGATTGGCAGCGTTTTAAAGATACCGTTGGCTTTGAACAGT
ATGTGTGTTATGAAGGCCAGATCGATTTTACCGAAAGCCTGCGTAGCAATAGCAATGTTGGTCTGCGTCGTGTTCGTGGTAGCGGTCGTGATC
TGTATCATCGTCTGCATGAAAATCCGCAGATGGAACAGGCATTCTATAAATACATGCGTAGCTGGTCTGAACTGGCAAATCAGCATCTGGTTG
AAGTTCTGGATCTGAGCGGCACCAGCAAACTGTTAGATTGTGGTGGTGGTGATGCAGTTAATAGCATTGCACTGGCACAGGCAAATCCGCAT
ATTGAAGCCGGTATTCTGGAAATTCCGCCTACCGCACCGCTGACCGAAAAGAAAATTGCAGAAGCAGGTCTGAGCGATCGTATTACCGTTAAA
CCGGGTGATATGCATACCGATGAATTTCCGACCGGCTATGATACCGTTATGTTTGCACATCAGCTGGTTATTTGGACACCGGAAGAAAATACC
GCACTGCTGCGTAAAGCATATAATGCACTGCCGGAAGGTGGTCGTGTTATTATCTTTAATTCCATGAGCAATGATGAAGGTGATGGTCCGGTT
GTTGCAGCACTGGATAGCGTTTATTTTGCAGCCCTGCCAGCCGAAGGTGGTATGATTTATAGCTGGGCAACCTATGAAGAAAGCCTGACCAAA
GCAGGTTTTAATCCGGAAACCTTTCAGCGCATTGATTTCCCTGGCTGGACACCGCATGGTGTGATTATTGCAACCAAATAA

Protein sequence of His$_6$-*Sa*OMT2 (41.3 kDa)

*MGSSHHHHHHSSGLVPRGSHMASMTGGQQMGRGSEL*MSCRTGTDTVPAGSHEQRTVESGEVMAKETTPRGGGVWAAADLLTPMAVRVAAT
LRLADHIAAGARTTEALAEAVGADRDALGRLLDHLVTAGVLSGTGPGAYDLTAMGRHLCEGAPEDMRAILDIEGALGHAELSLVHLLHTVRTGEAAF
PQQYGVTFWDDLSSDDGRAESFDTLMGARLTAHSPAVAGAYPWGTLRHVVDVGGGDGTMLIAILQSHPDLRGTVVDLPGPVRRAEKAIAAAGLD
HRADIAAGSFFDALPAGADGYLLSSILHNWDDASAARILRRCADAAQTTGRVLVVDYFGDRTVQTEGDLRMLGYFGGRQHTLEQLAELAGTVGLHT
TSVTPAGRYSVVELRAVG

Nucleotide sequence of *saomt2* (codon-optimized for *E. coli*)

ATGTCCTGTCGCACAGGTACCGATACAGTTCCCGCGGGTAGCCATGAGCAGCGGACGGTCGAATCTGGCGAAGTTATGGCCAAAGAGACCAC
GCCAAGAGGCGGCGGGGTTTGGGCTGCAGCAGACCTGTTAACTCCCATGGCGGTTCGCGTTGCGGCTACTTTGCGGCTCGCCGACCATATTG
CAGCAGGAGCACGGACTACAGAAGCACTTGCCGAGGCTGTGGGCGCAGACAGAGATGCGCTGGGACGACTCCTGGATCATTTAGTGACAGC
AGGAGTTCTTTCCGGGACCGGCCCAGGGGCCTACGATCTTACCGCGATGGGCCGGCACTTGTGCGAAGGGGCCCCCGAAGATATGAGAGCA
ATTTTAGATATAGAGGGTGCTTTGGGCCATGCCGAGTTGAGTCTTGTGCACCTTCTCCACACTGTGCGCACAGGAGAGGCTGCCTTCCCACAG
CAGTACGGAGTCACCTTCTGGGATGACTTGTCCTCTGACGACGGGCGTGCTGAGTCGTTTGATACATTAATGGGAGCACGGCTGACGGCGCA
CAGTCCCGCTGTAGCTGGTGCCTACCCCTGGGGTACACTGCGTCACGTAGTTGACGTCGGTGGTGGTGACGGTACCATGCTGATAGCTATCCT
GCAATCACATCCAGACCTTCGCGGGACCGTCGTAGACCTGCCTGGTCCTGTGCGCAGAGCTGAGAAAGCCATTGCAGCGGCAGGCCTGGATC
ATCGGGCTGACATTGCGGCCGGATCGTTCTTTGACGCCCTGCCTGCCGGAGCGGATGGCTACCTGCTTTCTAGCATCTTGCATAACTGGGACG
ATGCAAGTGCAGCCCGGATCCTGCGCCGGTGCGCCGACGCTGCACAGACAACAGGCCGGGTATTAGTGGTAGATTACTTTGGTGACCGGACT
GTACAAACGGAAGGCGATCTCCGTATGCTGGGATATTTCGGTGGGCGTCAGCACACCCTGGAACAATTAGCAGAGTTGGCCGGTACCGTCGG
TCTGCACACGACCAGCGTGACTCCGGCAGGTCGTTACTCAGTTGTGGAACTGCGCGCGGTGGGC

3. ^{1}H NMR chemical shifts of 4, 5, 7, 8, and 9

2-Acetylphloroglucinol (4)

^{1}H NMR (400 MHz, acetone-d_6): δ 2.58 (s, 3 H, CH_3), 5.91 (s, 2 H, H-4/6), 9.62 (1 H, OH), 11.91 (1 H, OH).

2-Acetyl-4-[^{13}C]methylphloroglucinol (5)

^{1}H NMR (400 MHz, acetone-d_6), δ 1.92 (d, $^{1}J_{CH}$ = 128 Hz, 3 H, $^{13}CH_3$), 2.57 (s, 3 H, CH_3), 6.09 (s, 1 H, H-6). The hydroxyl protons are too weak and cannot be assigned.

Kaempferol (7)

^{1}H NMR (400 MHz, acetone-d_6), δ 6.27–6.28 (m, 1H, H-6), 6.54–6.55 (m, 1H, H-8), 7.01–7.04 (AA' part of AA'BB', $^{3}J_{AB}$ = 8.95 Hz, 2H, H-3'/5'), 8.14–8.18 (BB' part of AA'BB', $^{3}J_{AB}$ = 8.95 Hz, 2H, H-2'/6'), 12.20 (s, 1H, OH).

6-[^{13}C]Methylkaempferol (8)

^{1}H NMR (400 MHz, acetone-d_6), δ 2.09 (d, $^{1}J_{CH}$ = 128 Hz, 3 H, $^{13}CH_3$), 6.63 (s, 1H, H-8), 12.41 (s, 1H, OH). Aromatic protons of ring B are overlaid by the signals of kaempferol (**7**).

7-O-[^{13}C]Methylkaempferol (9)

^{1}H NMR (400 MHz, acetone-d_6), δ 3.92 (d, $^{1}J_{CH}$ = 128 Hz, 3 H, $^{13}CH_3$), 6.33–6.34 (m, 1H, H-6), 6.67–6.72 (m, 1H, H-8), 7.01–7.04 (m, 2H, H-3'/5'), 8.16–8.20 (m, 2H, H-2'/6') 12.18 (1H, OH).

4. Supplementary Tables

Table S1: PCR conditions.

**Experiment**	**Initial denaturation**	**Thermal cycling**	**Final elongation**
Gene amplification	98 °C for 60 s	30 cycles of 98 °C for 10 s, 60 °C for 30 s, 72 °C for 50 s	72 °C for 60 s
Site-directed mutagenesis	98 °C for 60 s	30 cycles of 98 °C for 30 s, 60 °C for 30 s, 72 °C for 360 s	72 °C for 60 s

Table S2: Oligonucleotides used for gene amplification and site-directed mutagenesis.

**Oligonucleotide**	**Sequence**	**Target construct**
napB5_inf_for	TCGCGGATCCGAATTACTGACACCGGAAGCA	pET28a::*napB5*
napB5_inf_rev	CGGAGCTCGAATTTTATTAACCTGCACGTTTG	
napB5_Y136F_for	GGCAATCTGTTTGATGCCCTGGCCGAACATAAAG	pET28a::*napB5*_ Y136F
napB5_Y136F_rev	CAGGGCATCAAACAGATTGCCTTCATGACCATAC	
napB5_H248A_for	GCTGATTAAAGCATTCCTGCCGATGTTTG	pET28a::*napB5*_H248A
napB5_H248A_rev	CGGCAGGAATGCTTTAATCAGCACCACATC	
napB5_W149F_for	GCGAACCGTTCGCACGTCTGATGAATGGTG	pET28a::*napB5*_W149F
napB5_W149F_rev	CAGACGTGCGAACGGTTCGCGCAGATCTTTATG	
napB5_E347A_for	CTGCCGGTTGCAGCAGTTCTGAGCGCAAC	pET28a::*napB5*_E347A
napB5_E347A_rev	CAGAACTGCTGCAACCGGCAGTGCATGCG	
sfmM2_inf_for	TCGCGGATCCGAATTAATGACCATTAGCCTGGAAAATACC	pET28a::*sfmM2*
sfmM2_inf_rev	GACGGAGCTCGAATTTTATTTGGTTGCAATAATCACACC	
saomt2_inf_for	TCGCGGATCCGAATTAATGTCCTGTCGCACAGGTACC	pET28a::*saomt2*
saomt2_inf_rev	CGGAGCTCGAATTTTAGCCCACCGCGCGCAGTTCC	

Table S3: Conversion of flavonoids by NapB5 and product formation in extracted activity assays with purified enzyme and in situ generated [^{13}C-methyl]-*S*-adenosyl-L-methionine (SAM). Mean [%] ± standard deviation was calculated based on HPLC-DAD analysis using the ratio of area under the curve (AUC) to total of AUCs. The chemoselectivity of the methylation products was proven by ^{13}C NMR and HSQC experiments (Figures S18 and S20). nd: not determined.

Substrate	Detection wavelength	Total conversion	C-methylation	O-methylation	Dimethylation
kaempferol (**7**)	370 nm	45.3 ± 10.6	23.2 ± 9.47	20.6 ± 0.11	1.51 ± 1.05
luteolin	350 nm	39.7 ± 5.35	36.8 ± 5.04	2.87 ± 0.31	-
quercetin	370 nm	31.6 ± 4.97	29.3 ± 4.70	2.35 ± 0.27	-
chrysin	280 nm	8.67 ± 4.38	8.24 ± 4.14	0.43 ± 0.24	-
daidzein	280 nm	3.15 ± 1.58	-	3.15 ± 1.58	-
baicalein	322 nm	-	-	-	-
noreugenin	280 nm	1.09 ± 0.22	nd	nd	nd
taxifolin	280 nm	20.5 ± 1.18	nd	nd	nd
eriodictyol	280 nm	93.2 ± 0.09	nd	nd	nd

Table S4: Conversion of kaempferol (**7**) by NapB5 and variants and product formation in extracted activity assays with cell-free lysate and in situ generated [^{13}C-methyl]-*S*-adenosyl-L-methionine (SAM). Mean [%] ± standard deviation was calculated based on HPLC-DAD analysis at 370 nm using the ratio of area under the curve (AUC) to total of AUCs.

Lysate	Total conversion	Formation of 6-methylkaempferol (**8**)	Formation of 7-O-methylkaempferol (**9**)	Formation of 6-methyl-7-O-methylkaempferol (**10**)
empty vector	0.07	0.02	0.05	-
NapB5	78.9 ± 1.43	61.3 ± 0.89	14.5 ± 0.17	3.07 ± 0.39
H248A	1.90 ± 0.06	1.32 ± 0.06	0.45 ± 0.04	0.13 ± 0.04
Y136F	1.22 ± 0.07	0.20 ± 0.01	1.00 ± 0.07	0.02 ± 0.01
W149F	13.9 ± 0.39	0.31 ± 0.01	13.8 ± 0.38	0.01 ±0.01
E347A	6.18 ± 0.15	5.23 ± 0.13	0.91 ± 0.02	0.04 ± 0.01

5. Supplementary Figures

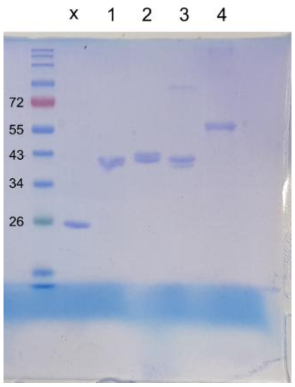

Figure S1: SDS-PAGE of His$_6$-tagged MTs and bovine serum albumin (BSA) standard (1 mg·mL^{-1}) after Ni-NTA purification. Ladder: NEB Color Prestained Protein Standard. X: enzyme not used in this study. 1: SfmM2 (43.8 kDa). 2: NapB5 (43.2 kDa). 3: *Sa*OMT2 (41.3 kDa). 4: BSA standard (66.5 kDa).

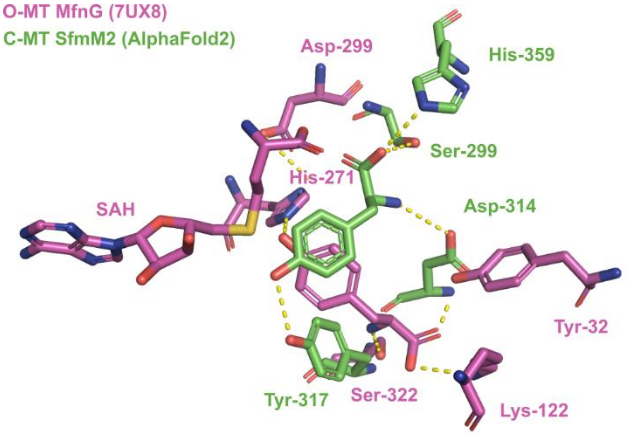

Figure S2: Active site of superimposed tyrosine O-MT MfnG (magenta; co-crystallized with SAH and soaked with L-tyrosine; PDB: 7UX8) and tyrosine C-MT SfmM2 (green; AlphaFold2; docked with L-tyrosine). The respective acceptor nucleophiles are located near the sulfur of SAH.

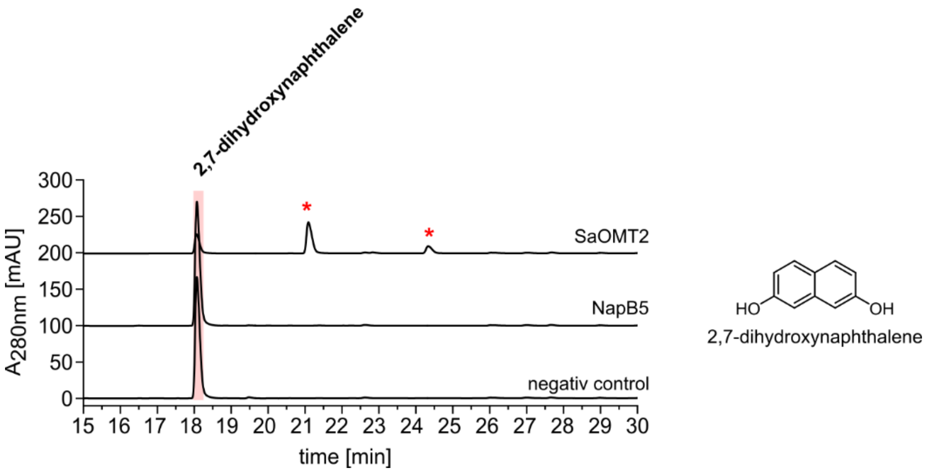

Figure S3: HPLC-DAD chromatograms of NapB5 and *Sa*OMT2 activity assay with 2,7-dihydroxynaphthalene using an in situ *S*-adenosyl-L-methionine (SAM) supply cascade with *Ec*MAT and *Ec*MTAN. No conversion by NapB5 was observed. Putative O-methylated products of *Sa*OMT2 are marked with an asterisk.

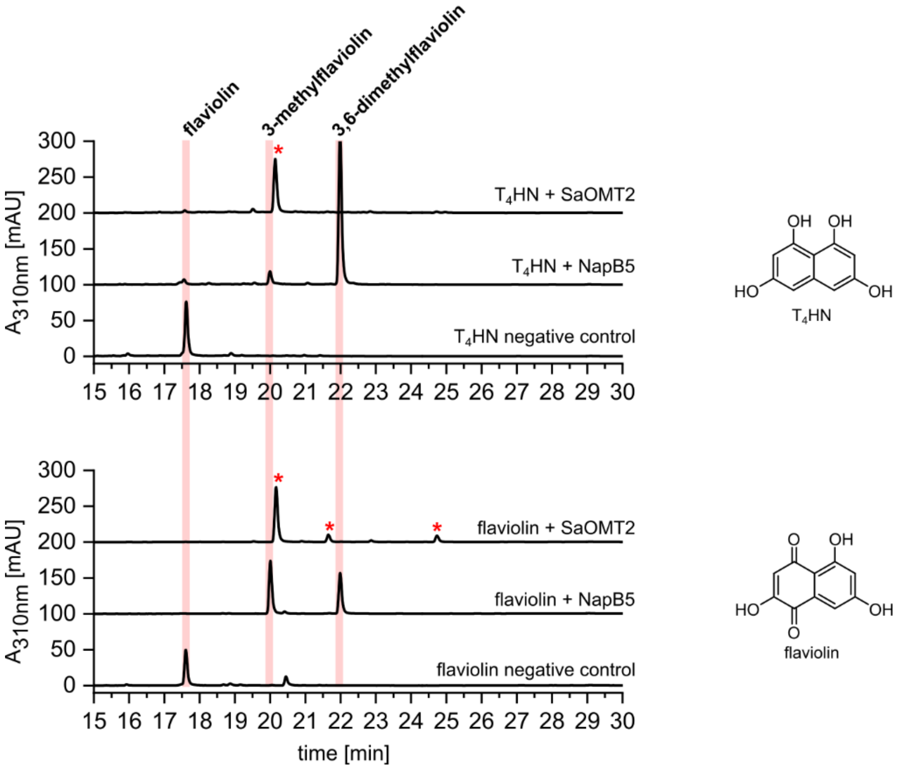

Figure S4: HPLC-DAD chromatograms of NapB5 and *Sa*OMT2 activity assay with 1,3,6,8-tetrahydroxynaphthalene (T_4HN) or flaviolin using an in situ *S*-adenosyl-L-methionine (SAM) supply cascade with *Ec*MAT and *Ec*MTAN. Putative O-methylated products of *Sa*OMT2 are marked with an asterisk and show distinct retention times compared to the C-methylated products of NapB5 (3-methylflaviolin and 3,6-dimethylflaviolin), which have been characterized in our previous study.[1]

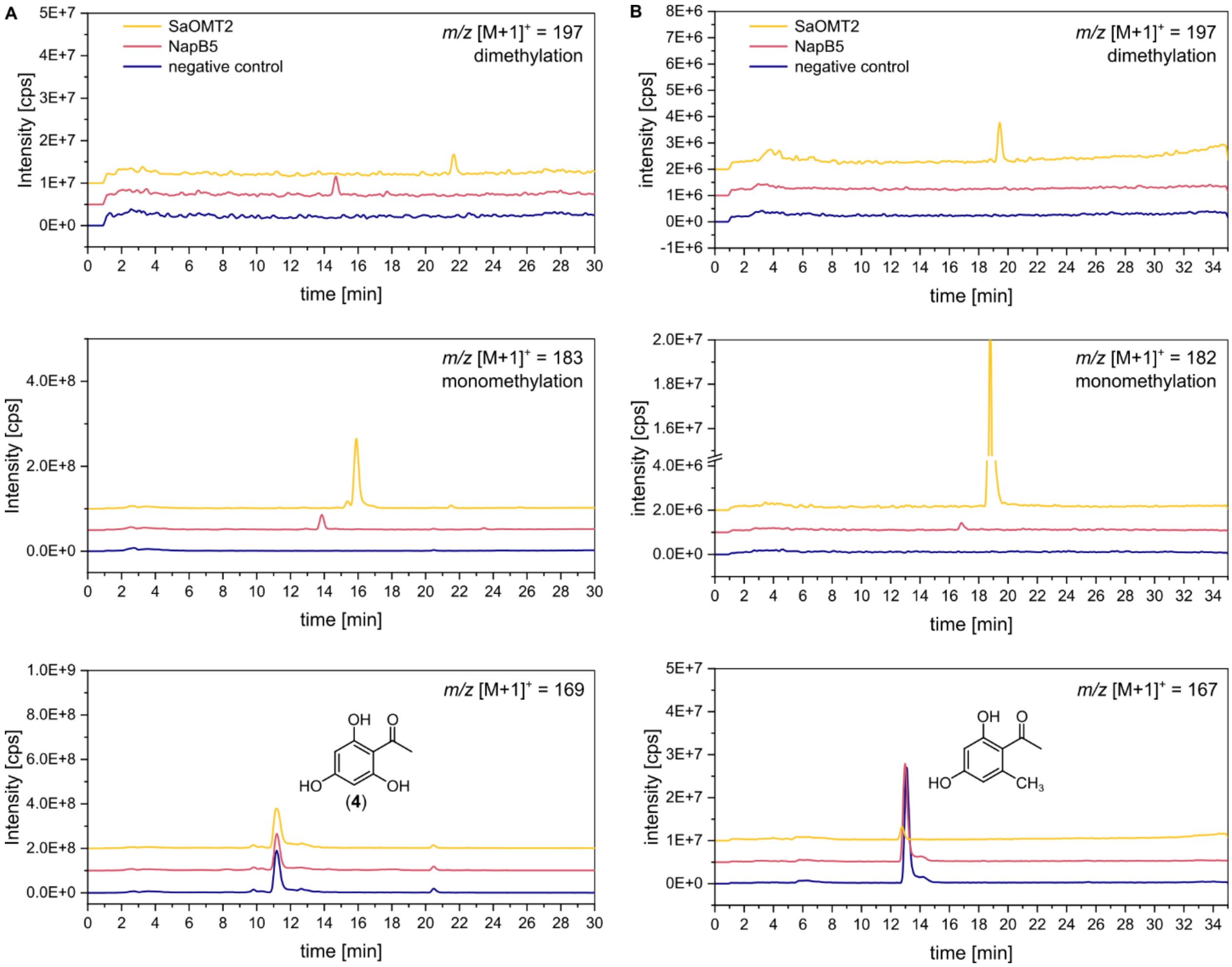

Figure S5: Extracted ion chromatograms (HPLC-MS) of NapB5 and *Sa*OMT2 activity assays with: **A**) 2-acetylphloroglucinol (**4,** *m/z* [M+1]$^+$ = 169) using an in situ *S*-adenosyl-L-methionine (SAM) supply cascade with *Ec*MAT and *Ec*MTAN. The *m/z* values of substrate ([M+1]$^+$), mono- (+14), and dimethylation (+28) are shown; **B**) 2,4-dihydroxy-6-methylacetophenone (*m/z* [M+1]$^+$ = 167) using an in situ [^{13}C-methyl]-SAM supply cascade with *Ec*MAT and *Ec*MTAN. The *m/z* values of substrate ([M+1]$^+$), mono- (+15), and dimethylation (+30) are shown.

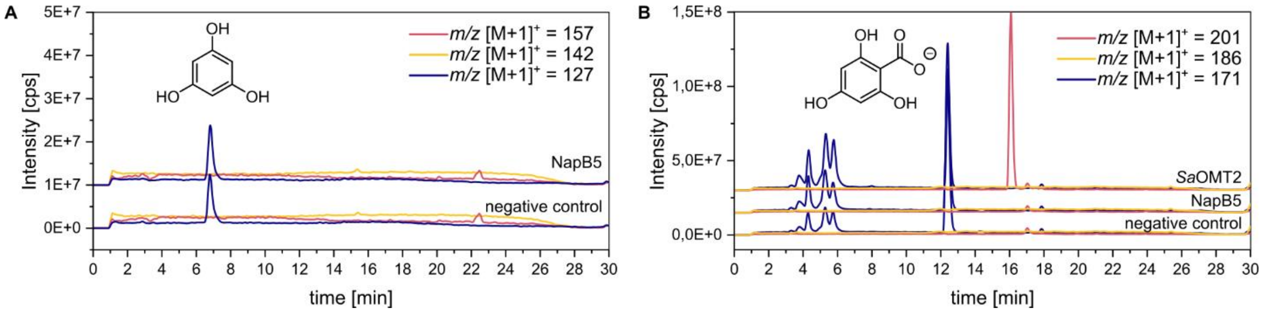

Figure S6: Extracted ion chromatograms (HPLC-MS) of NapB5 activity assays with: **A**) phloroglucinol (*m/z* [M+1]$^+$ = 127); **B**) 2,4,6-trihydroxybenzoic acid (*m/z* [M+1]$^+$ = 171) using an in situ [^{13}C-methyl]-SAM supply cascade with *Ec*MAT and *Ec*MTAN. The *m/z* values of substrate ([M+1]$^+$), mono- (+15), and dimethylation (+30) are shown.

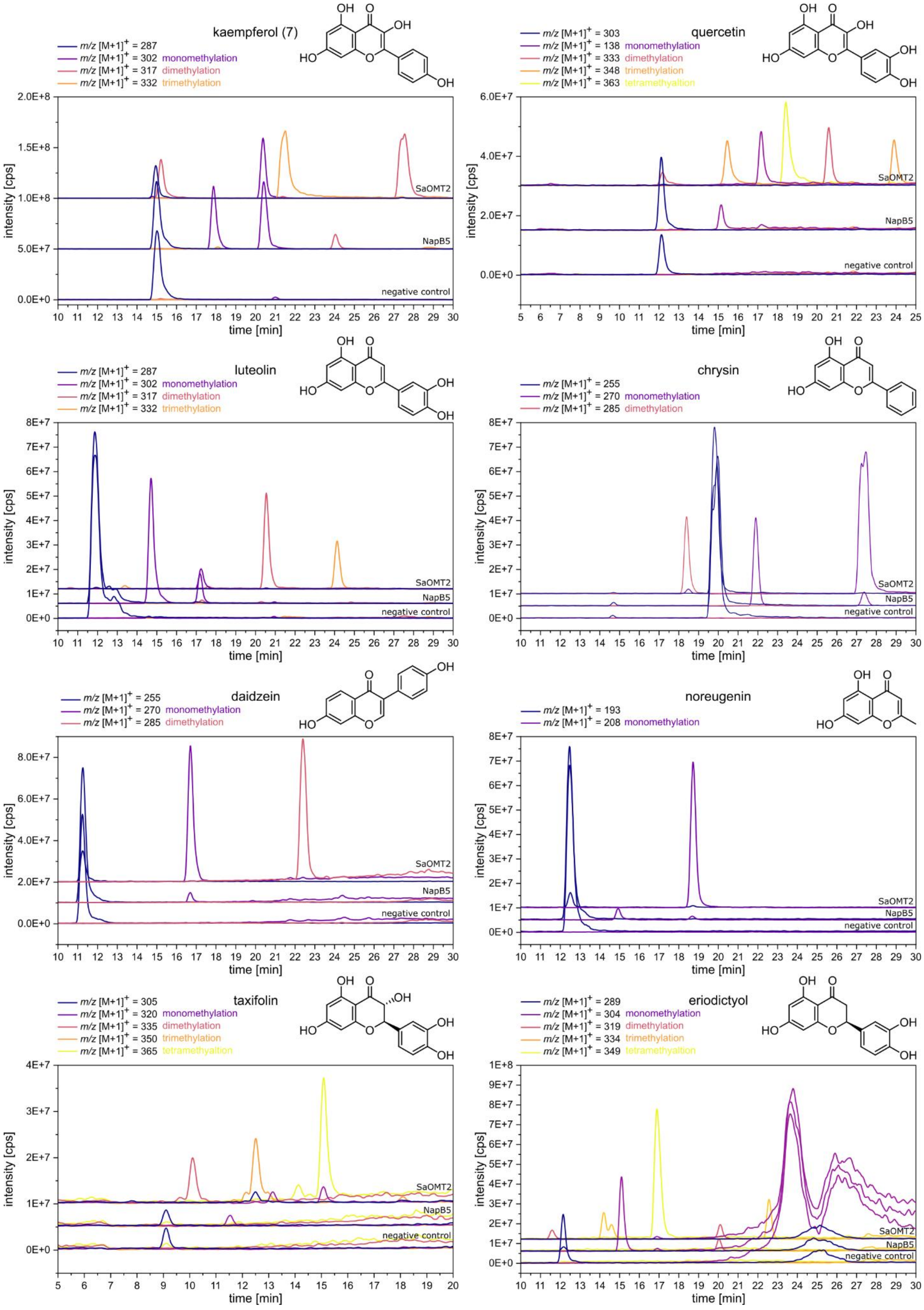

kaempferol (7)
m/z [M+1]+ = 287
m/z [M+1]+ = 302 monomethylation
m/z [M+1]+ = 317 dimethylation
m/z [M+1]+ = 332 trimethylation
quercetin
m/z [M+1]+ = 303
m/z [M+1]+ = 138 monomethylation
m/z [M+1]+ = 333 dimethylation
m/z [M+1]+ = 348 trimethylation
m/z [M+1]+ = 363 tetramethyaltion
luteolin
m/z [M+1]+ = 287
m/z [M+1]+ = 302 monomethylation
m/z [M+1]+ = 317 dimethylation
m/z [M+1]+ = 332 trimethylation
chrysin
m/z [M+1]+ = 255
m/z [M+1]+ = 270 monomethylation
m/z [M+1]+ = 285 dimethylation
daidzein
m/z [M+1]+ = 255
m/z [M+1]+ = 270 monomethylation
m/z [M+1]+ = 285 dimethylation
noreugenin
m/z [M+1]+ = 193
m/z [M+1]+ = 208 monomethylation
taxifolin
m/z [M+1]+ = 305
m/z [M+1]+ = 320 monomethylation
m/z [M+1]+ = 335 dimethylation
m/z [M+1]+ = 350 trimethylation
m/z [M+1]+ = 365 tetramethyaltion
eriodictyol
m/z [M+1]+ = 289
m/z [M+1]+ = 304 monomethylation
m/z [M+1]+ = 319 dimethylation
m/z [M+1]+ = 334 trimethylation
m/z [M+1]+ = 349 tetramethyaltion
SaOMT2
NapB5
negative control
intensity [cps]
time [min]
OH
HO
O

↑ **Figure S7**: Extracted ion chromatograms (HPLC-MS) of NapB5 and *Sa*OMT2 activity assays with flavonoids using an in situ [^{13}C-methyl]-*S*-adenosyl-L-methionine (SAM) supply cascade with *Ec*MAT and *Ec*MTAN. The *m/z* values of the substrate ($[M+1]^+$) and mono-/di-/tri-/tetramethylation are shown. O-methylated products by NapB5 appear as identical peaks in activity assays with the O-MT *Sa*OMT2.

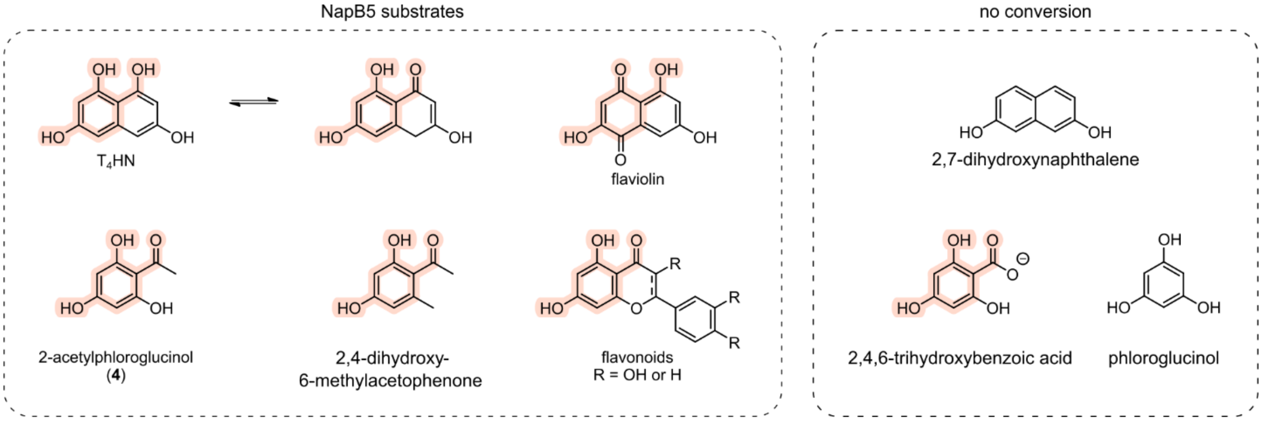

Figure S8: Compounds tested in NapB5 activity assays. The 2,4-dihydroxyacetophenone pattern favorable for substrate binding is highlighted.

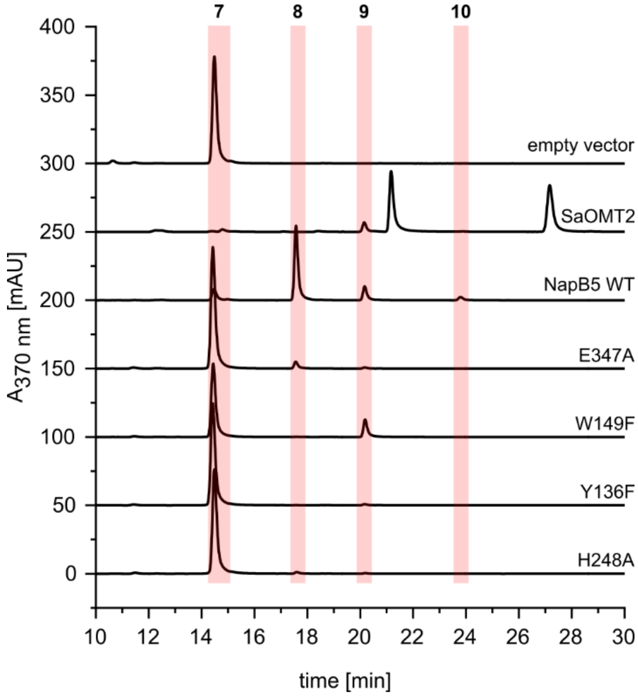

Figure S9: HPLC-DAD chromatograms (370 nm) of *Sa*OMT2, NapB5 wildtype (WT) and NapB5 variants activity assays with kaempferol (**7**) using an in situ *S*-adenosyl-L-methionine (SAM) supply cascade with *Ec*MAT and *Ec*MTAN. NapB5 WT and NapB5 variants assays were performed with cell-free lysate of *E. coli* BL21Gold (DE3) cells transformed with the respective constructs. Cell-free lysate of *E. coli* BL21Gold (DE3) cells transformed with the empty vector pET28a was used for the negative control.

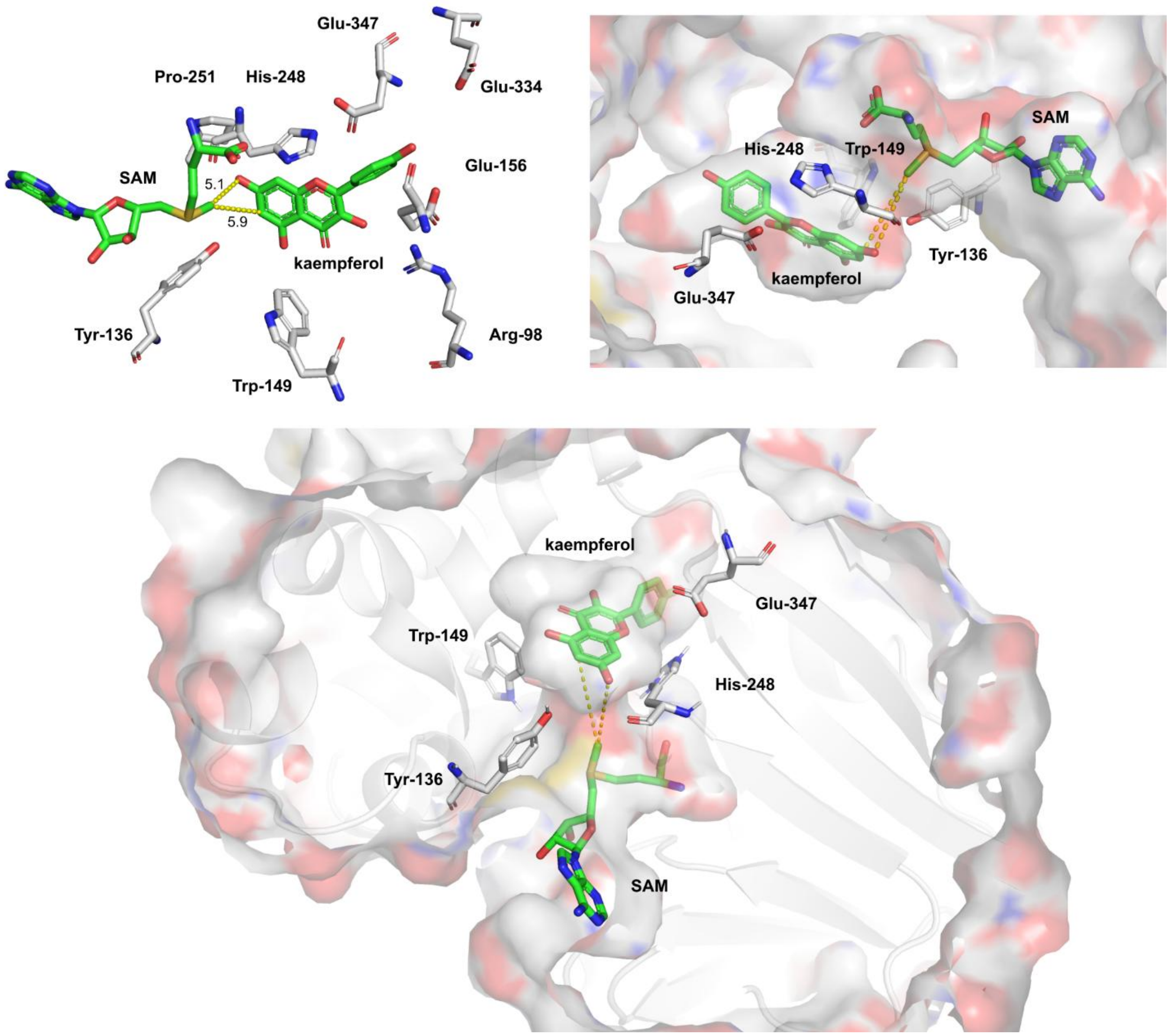

Figure S10: Docking of kaempferol (**7**) and *S*-adenosyl-L-methionine (SAM) into an AlphaFold2 model of NapB5.

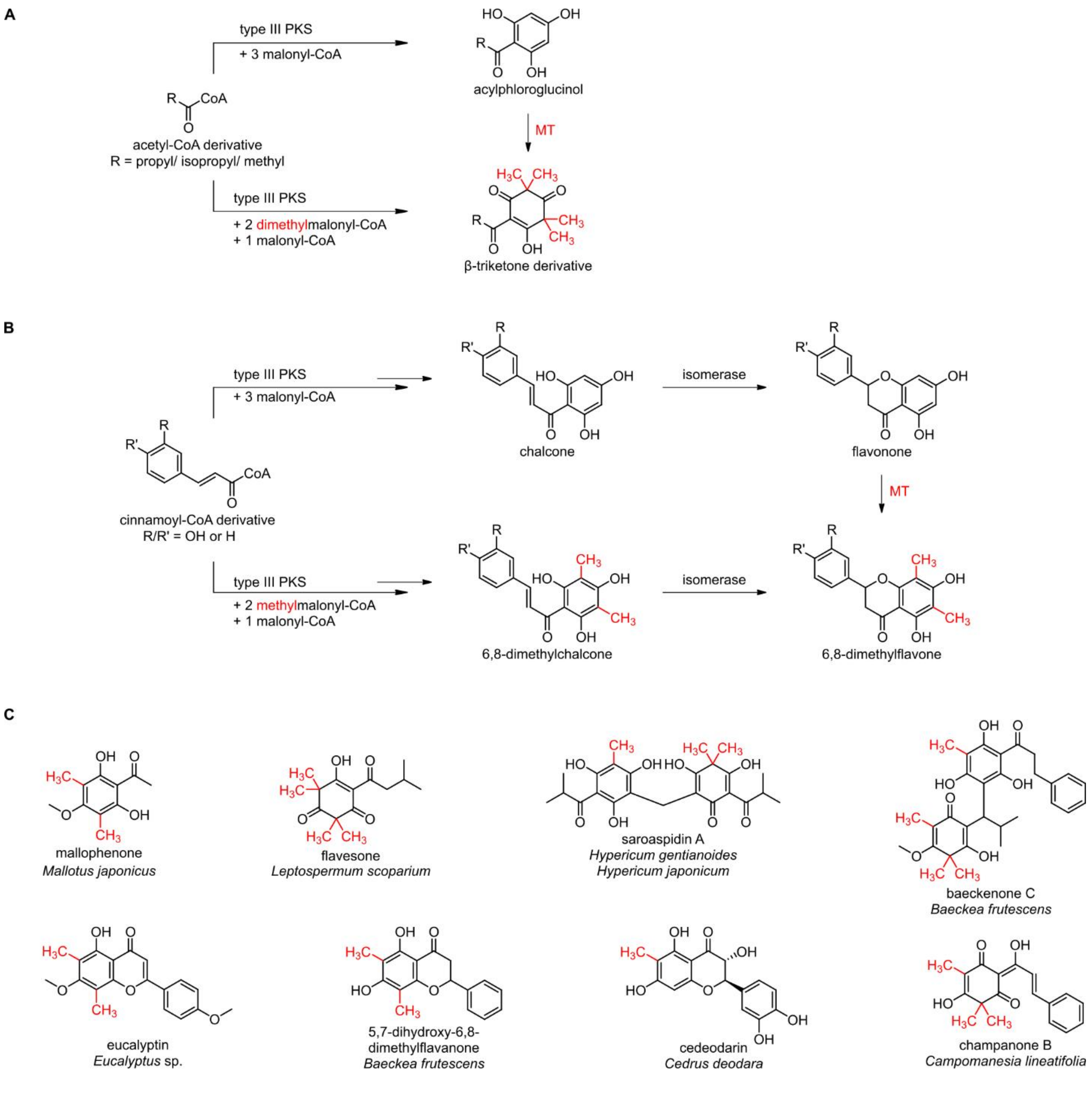

Figure S11: **A**) Proposed biosynthetic pathways of C-methylated acylphloroglucinols in plants. **B**) Proposed biosynthetic pathways of C-methylated chalcones and flavonoids in plants. **C**) C-methylated acylphloroglucinol, chalcone, and flavonoid natural products.

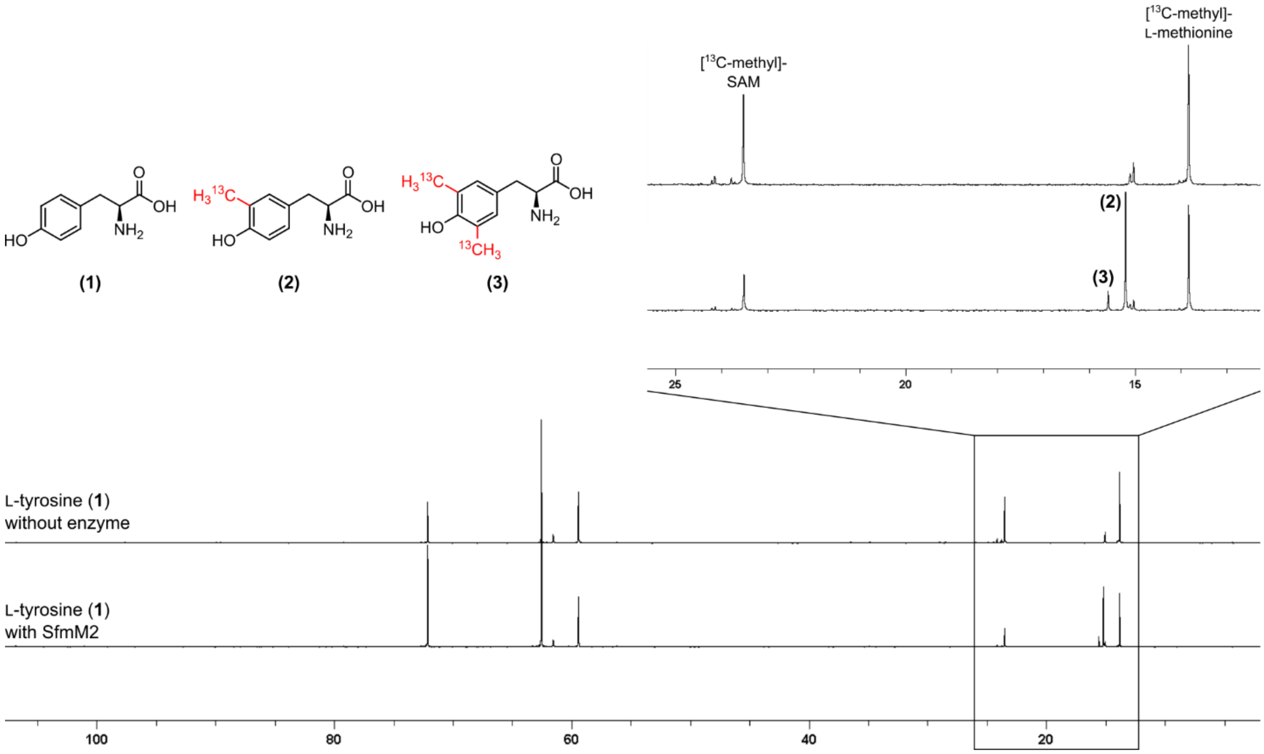

Figure S12: ^{13}C NMR spectra (D_2O) of SfmM2 activity assays with L-tyrosine (**1**) and negative control without enzyme. The signals of the ^{13}C-labeled methyl groups of [^{13}C-methyl]-L-methionine, [^{13}C-methyl]-SAM, **2**, and **3** are assigned.

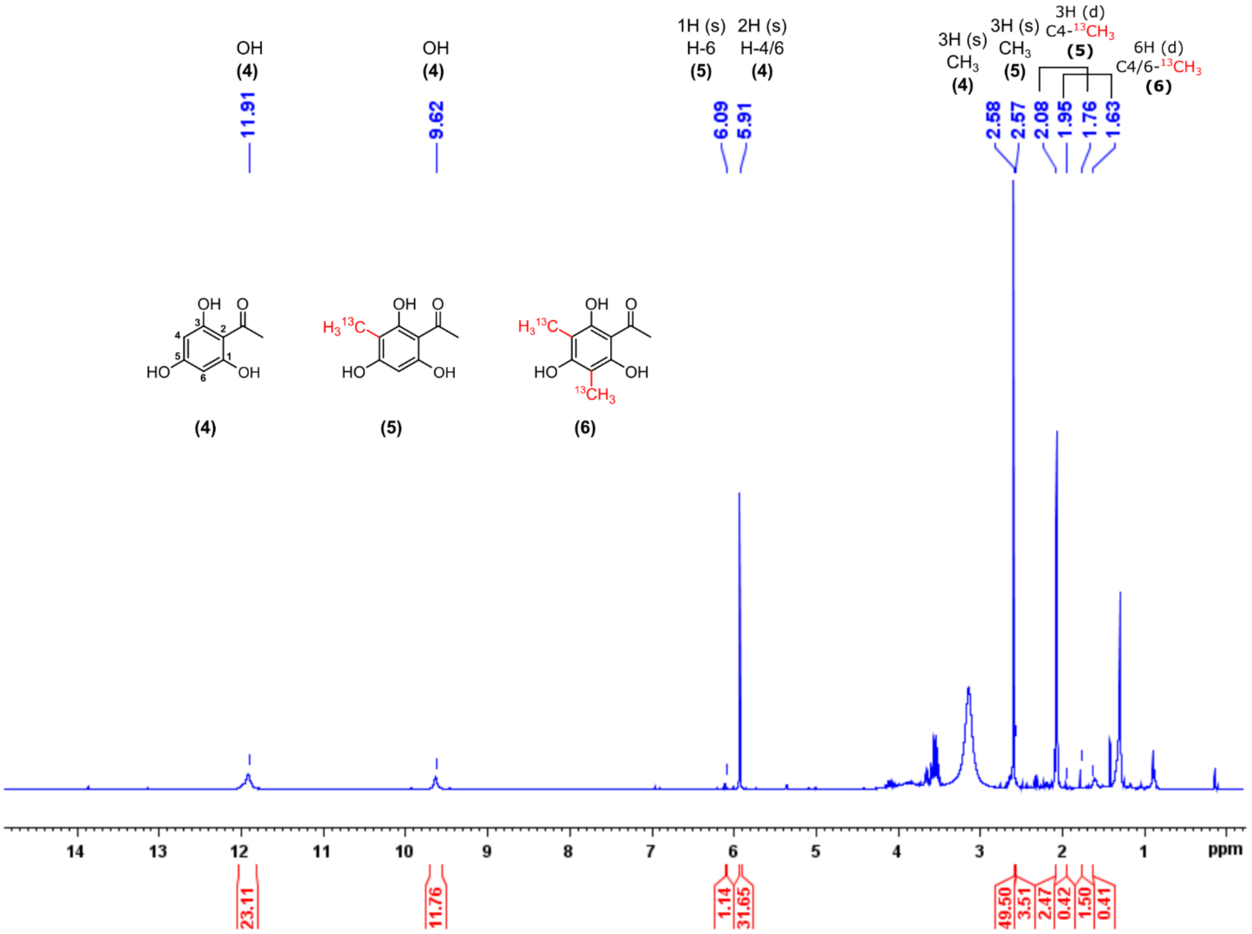

Figure S13: ^{1}H NMR spectrum (acetone-d_6) of extracted NapB5 assays with 2-acetylphloroglucinol (**4**). The chemical shifts of **4** and **5** are listed on page 7.

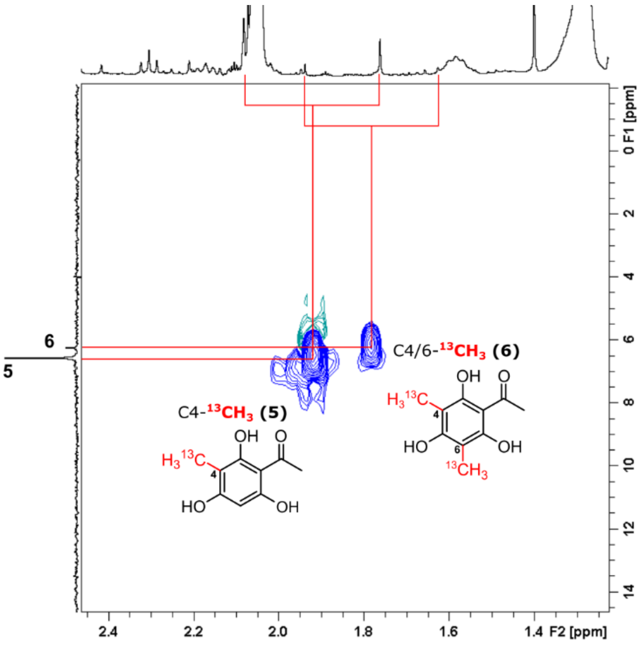

Figure S14: HSQC spectrum (acetone-d_6) of extracted NapB5 assays with 2-acetylphloroglucinol (**4**). The signals of the ^{13}C-labeled methyl groups of **5** and **6** correlate with the $^{1}J_{CH}$ doublets of the ^{1}H spectrum.

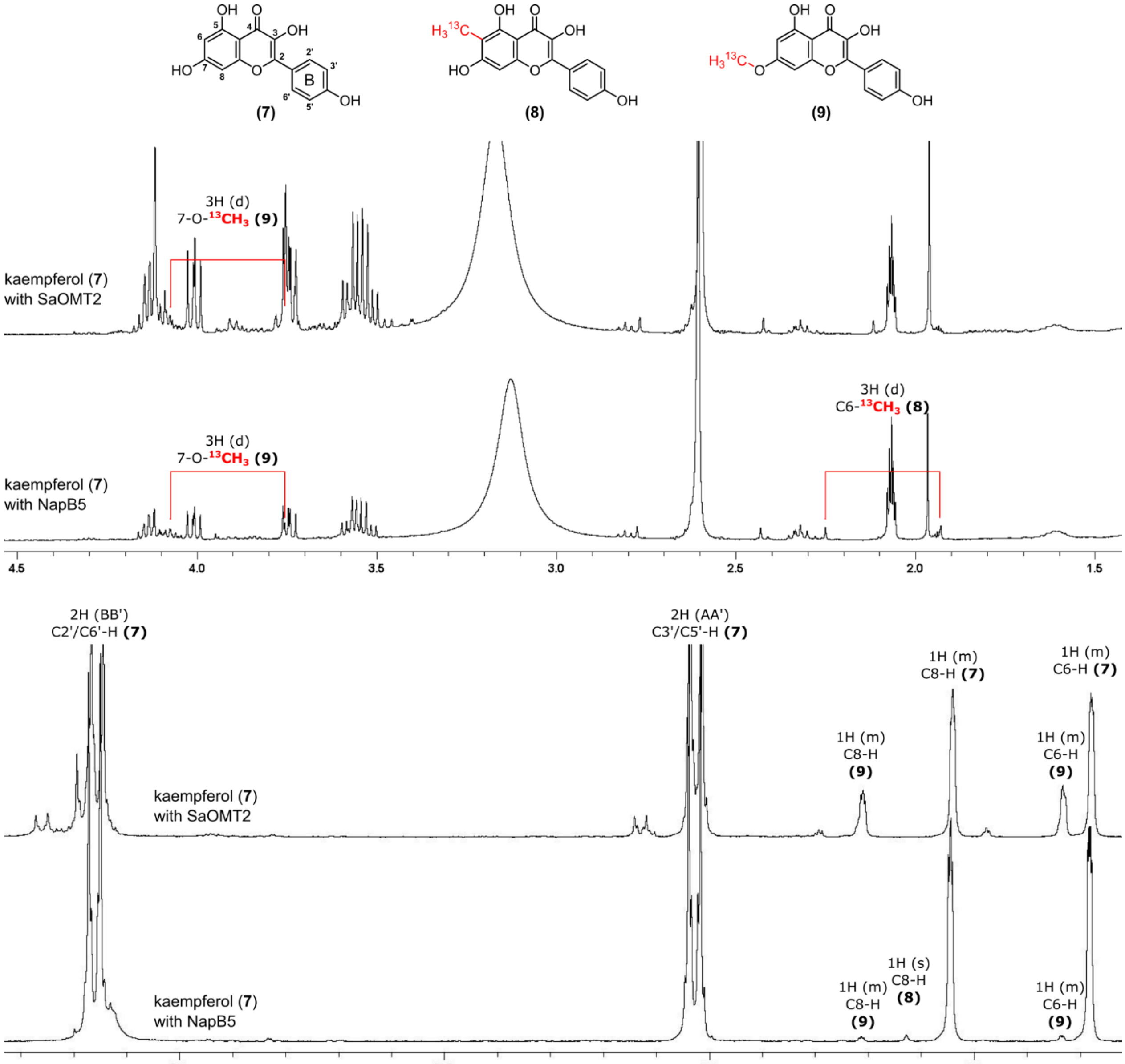

Figure S15: Comparison of ^{1}H NMR spectra (acetone-d_6) of extracted NapB5 and *Sa*OMT2 activity assays with kaempferol (**7**). The protons of the ^{13}C-labeled methyl groups appear as doublet with a heteronuclear coupling ($^{1}J_{CH}$) constant of 128 Hz. The signals of the aromatic protons of ring B appear as an AA'BB' system. The chemical shifts of **7**, **8** and **9** are listed on page 7.

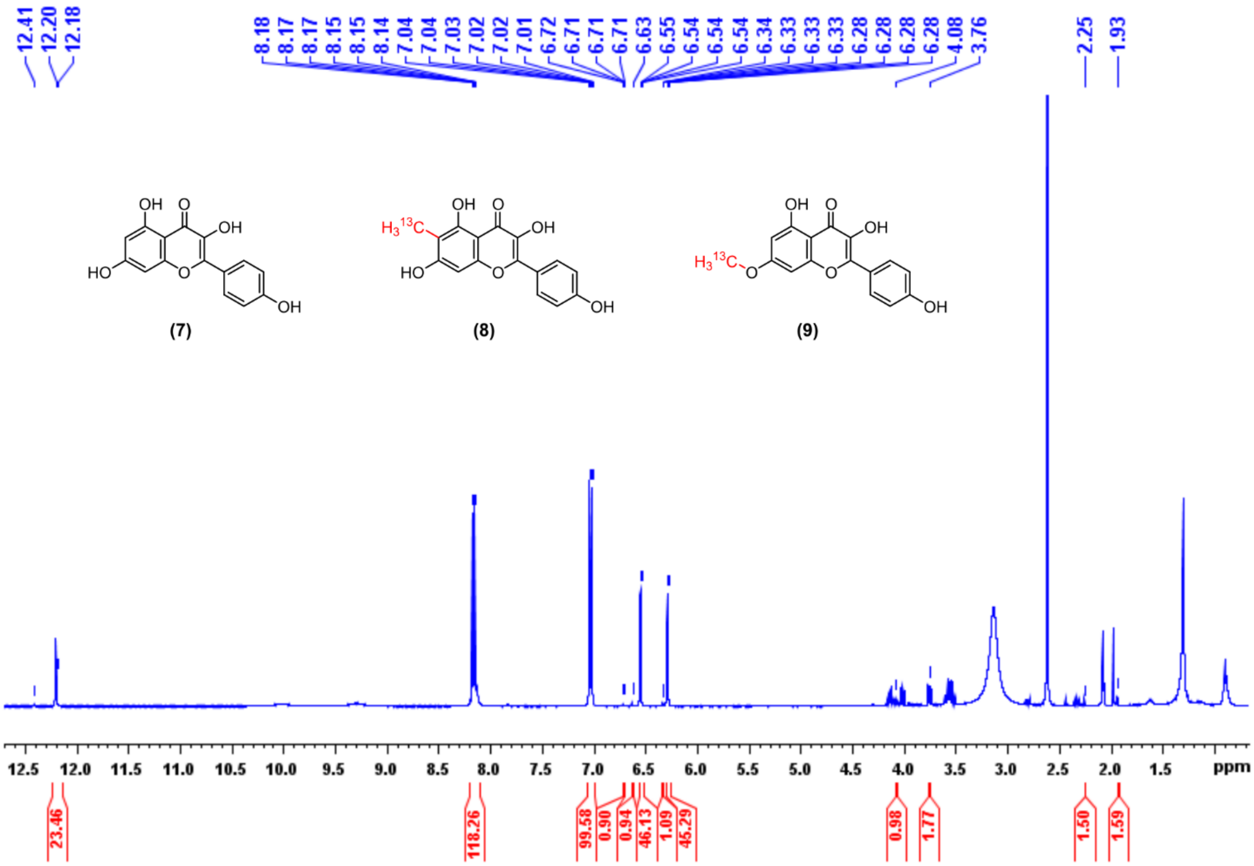

Figure S16: ^{1}H NMR spectrum (acetone-d_6) of extracted NapB5 assays with kaempferol (**7**). The chemical shifts of **7**, **8** and **9** are assigned in Figure S15 and listed on page 7.

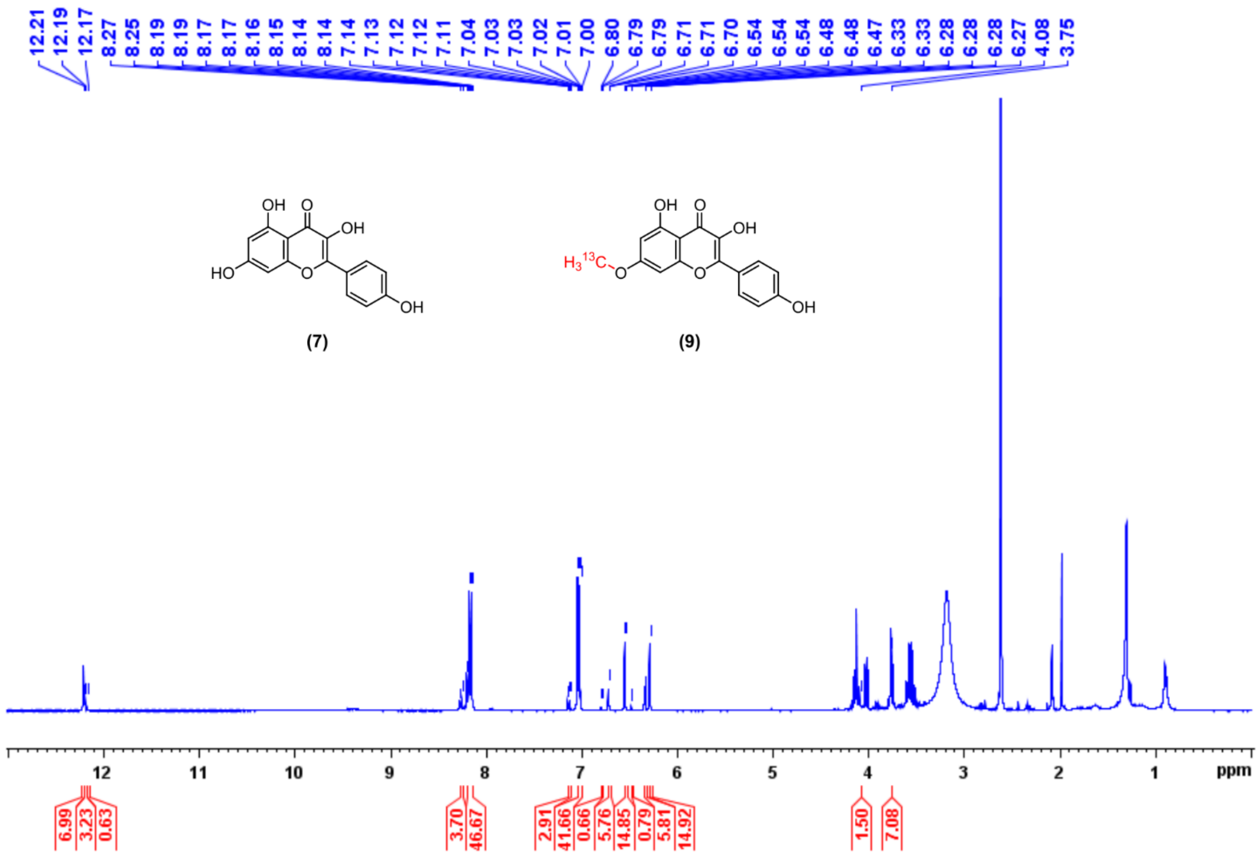

Figure S17: ^{1}H NMR spectrum (acetone-d_6) of extracted *Sa*OMT2 assays with kaempferol (**7**) yielding **9** and other O-methylated products. The chemical shifts of **7** and **9** are assigned in Figure S15 and listed on page 7.

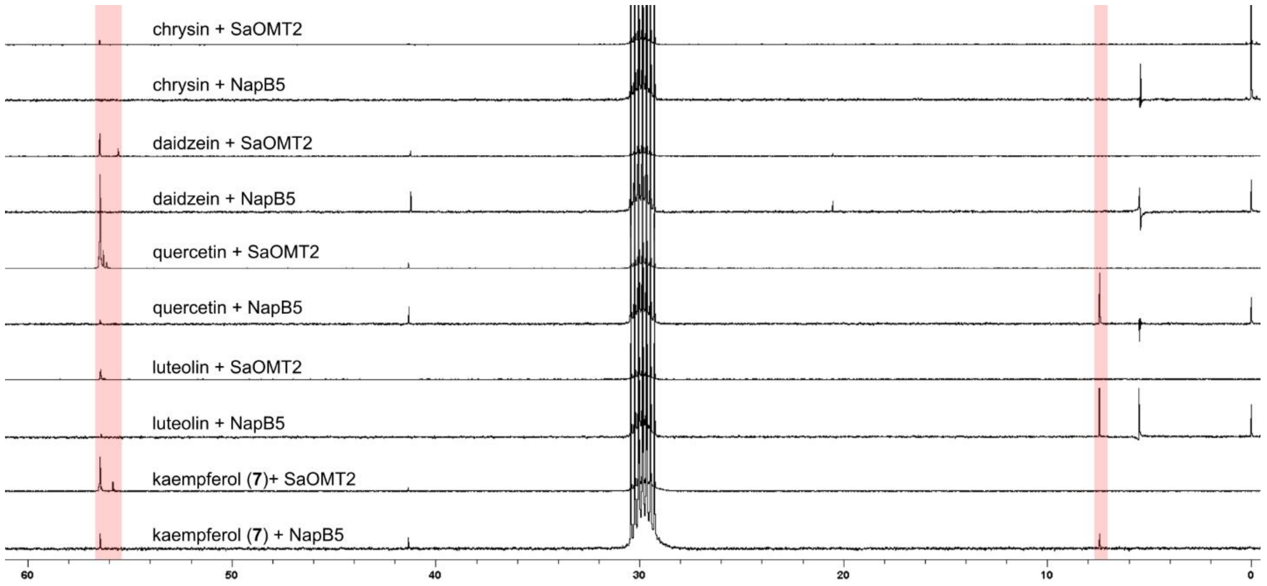

Figure S18: ^{13}C NMR spectra (acetone-d_6) of extracted NapB5 and *Sa*OMT2 activity assays with flavonoids. Due to low substrate concentrations, only ^{13}C-labeled methyl signals are visible. Signals of O-methylation appear at 55–57 ppm and signals of C-methylation at 7.5 ppm. For samples of luteolin with NapB5, quercetin with NapB5, daidzein with NapB5, and chrysin with NapB5 acetone-d_6 with 0.03% TSM was used as solvent. The conversion of daidzein and chrysin by NapB5 is too low to be detectable in the ^{13}C NMR spectra, but the corresponding signals are visible in the HSQC spectra (Figure S20 C and D).

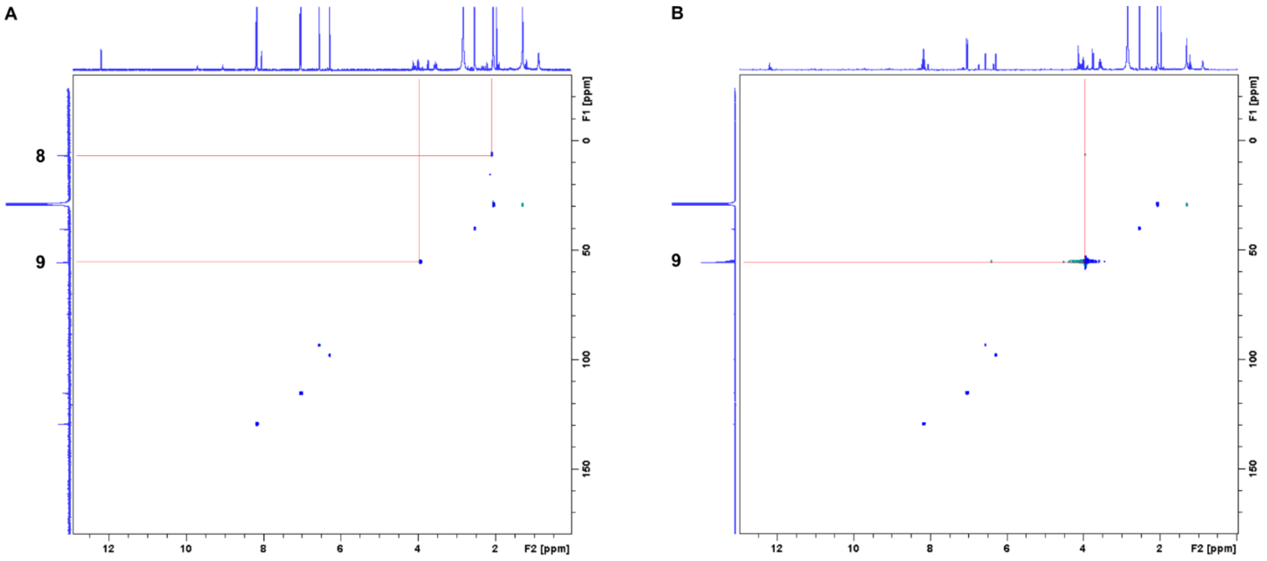

Figure S19: HSQC spectra (acetone-d_6) of: **A**) extracted NapB5 assays with kaempferol (**7**); **B**) extracted *Sa*OMT2 assays with **7**.

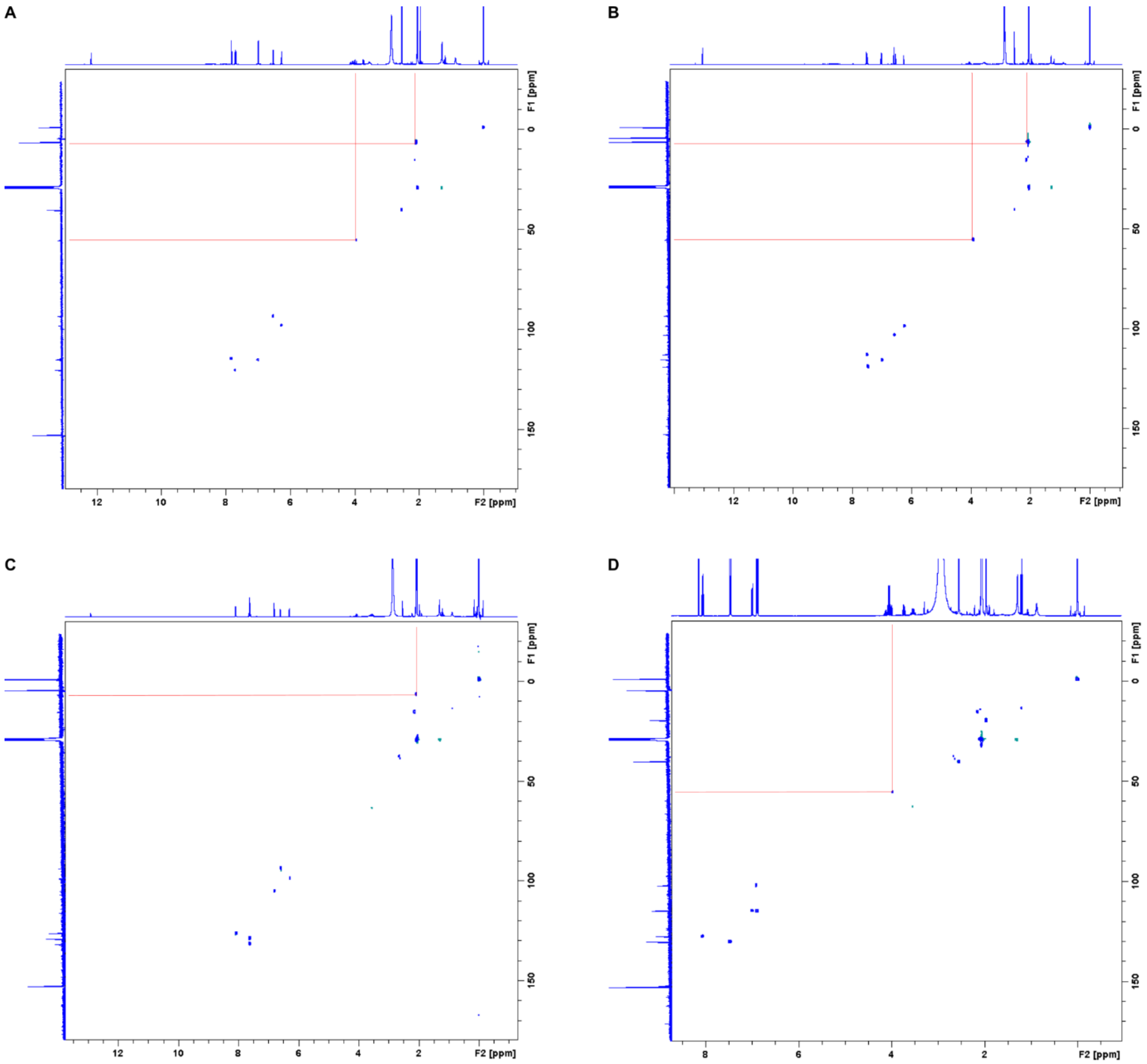

Figure S20: HSQC spectra (acetone-d_6 with 0.03% TSM) of extracted NapB5 assays with **A**) quercetin; **B**) luteolin; **C**) chrysin; **D**) daidzein.

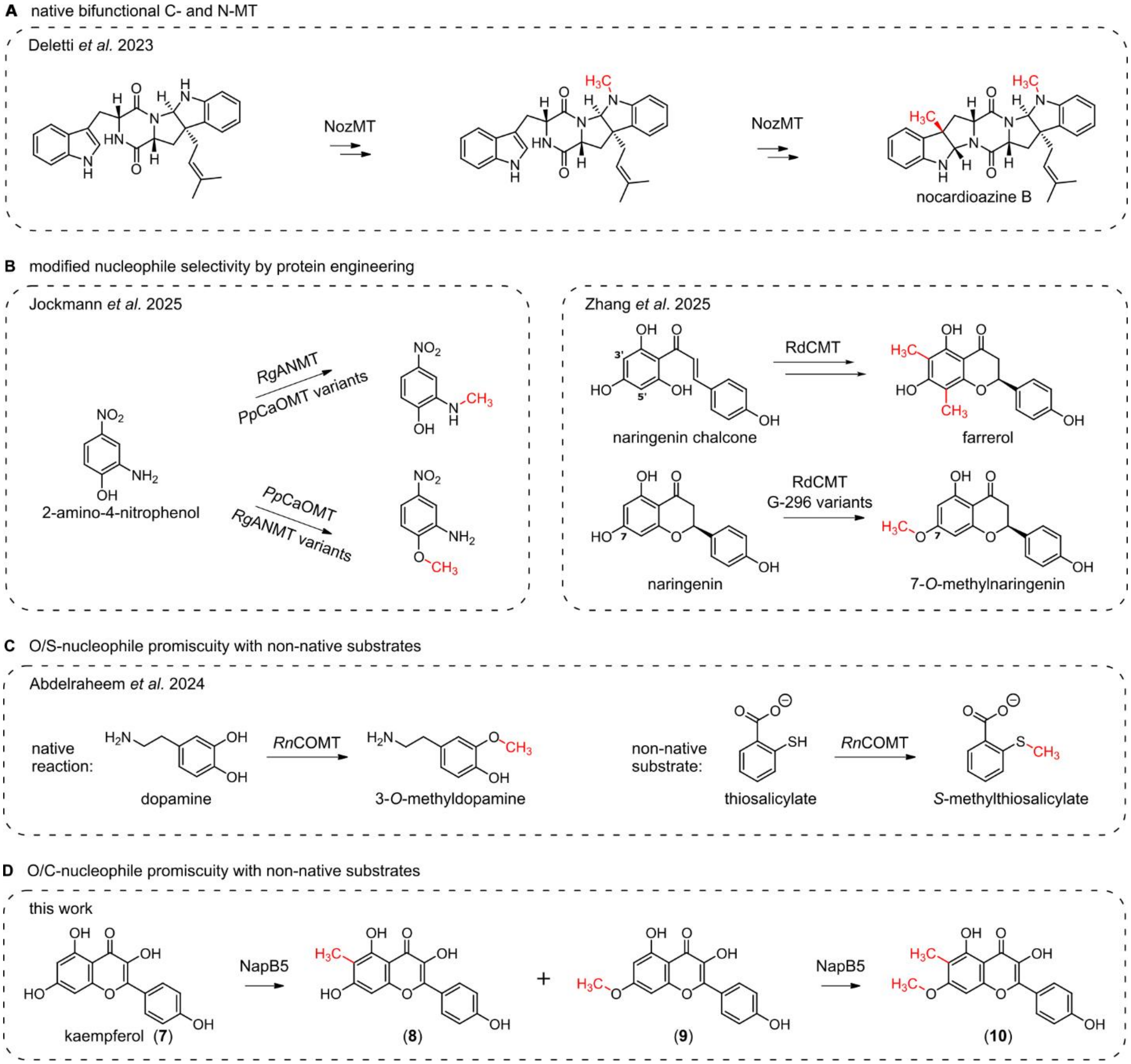

Figure S21: Native and modulated nucleophile selectivity of MTs. **A**) Native bifunctional MT NozMT.[8] **B**–**D**) Induced modulation of chemoselectivity by protein engineering of *Rg*ANMT, *Pp*CaOMT,[9] and *Rd*CMT[10] (**B**), or by substrate choice for *Rn*COMT[11] (**C**) and NapB5 (**D**).

6. Supplementary References

[1] J. Breiltgens, A. Paul, Z. Zou, J. N. Andexer, M. Müller, *J. Nat. Prod.* **2025**, *88*, 2750–2756.

[2] J. Siegrist, S. Aschwanden, S. Mordhorst, L. Thöny-Meyer, M. Richter, J. N. Andexer, *ChemBioChem* **2015**, *16*, 2576–2579.

[3] S. Mordhorst, J. Siegrist, M. Müller, M. Richter, J. N. Andexer, *Angew. Chem. Int. Ed.* **2017**, *56*, 4037–4041.

[4] F. Madeira, N. Madhusoodanan, J. Lee, A. Eusebi, A. Niewielska, A. R. N. Tivey, R. Lopez, S. Butcher, *Nucleic. Acids Res.* **2024**, *52*, W521-W525.

[5] M. Mirdita, K. Schütze, Y. Moriwaki, L. Heo, S. Ovchinnikov, M. Steinegger, *Nat. Methods* **2022**, *19*, 679–682.

[6] J. Jumper, R. Evans, A. Pritzel, T. Green, M. Figurnov, O. Ronneberger, K. Tunyasuvunakool, R. Bates, A. Žídek, A. Potapenko, A. Bridgland, C. Meyer, S. A. A. Kohl, A. J. Ballard, A. Cowie, B. Romera-Paredes, S. Nikolov, R. Jain, J. Adler, T. Back, S. Petersen, D. Reiman, E. Clancy, M. Zielinski, M. Steinegger, M. Pacholska, T. Berghammer, S. Bodenstein, D. Silver, O. Vinyals, A. W. Senior, K. Kavukcuoglu, P. Kohli, D. Hassabis, *Nature* **2021**, *596*, 583–589.

[7] G. M. Morris, R. Huey, W. Lindstrom, M. F. Sanner, R. K. Belew, D. S. Goodsell, A. J. Olson, *J. Comput. Chem.* **2009**, *30*, 2785–2791.

[8] G. Deletti, S. D. Green, C. Weber, K. N. Patterson, S. S. Joshi, T. M. Khopade, M. Coban, J. Veek-Wilson, T. R. Caulfield, R. Viswanathan, A. L. Lane, *Nat. Commun.* **2023**, *14*, 2558.

[9] E. Jockmann, H. Girame, W. Steinchen, K. Kind, G. Bange, K. Tittmann, M. Müller, F. Feixas, M. Garcia-Borràs, J. N. Andexer, *ACS Catal.* **2025**, 6410–6425.

[10] M. Zhang, Y.-O. Bao, Z. Dai, Z. Qian, H. Yu, J.-J. Zhou, Y. Chen, Z. Wang, K. Wang, M. Cai, M. Ye, *J. Am. Chem. Soc.* **2025**, *147*, 17132–17143.

[11] E. Abdelraheem, E. Jockmann, J. Li, S. Günther, J. N. Andexer, P.-L. Hagedoorn, U. Hanefeld, *ChemCatChem* **2024**, *16*, e202301217.
